# Improved Active Disturbance Rejection Control for Trajectory Tracking Control of Lower Limb Robotic Rehabilitation Exoskeleton

**DOI:** 10.3390/s20133681

**Published:** 2020-06-30

**Authors:** Sumit Aole, Irraivan Elamvazuthi, Laxman Waghmare, Balasaheb Patre, Fabrice Meriaudeau

**Affiliations:** 1 Department of Instrumentation Engineering, Shri Guru Gobind Singhji Institute of Engineering and Technology, Nanded 431606, Maharashtra, India; sumit.aole@gmail.com (S.A.); lmwaghmare@sggs.ac.in (L.W.); bmpatre@sggs.ac.in (B.P.); 2 Department of Electrical & Electronic Engineering, Universiti Teknologi PETRONAS, Seri Iskandar 32610, Perak Darul Ridzuan, Malaysia; 3 ImViA, University of Burgundy, Maison de l’université, 21078 Dijon, Le Creusot, France; fabrice.meriaudeau@u-bourgogne.fr

**Keywords:** improved active disturbance rejection control (I-ADRC), lower limb robotic rehabilitation exoskeleton (LLRRE), trajectory tracking, linear extended state observer (LESO), tracking differentiator (TD), nonlinear state error feedback (NLSEF)

## Abstract

Neurological disorders such as cerebral paralysis, spinal cord injuries, and strokes, result in the impairment of motor control and induce functional difficulties to human beings like walking, standing, etc. Physical injuries due to accidents and muscular weaknesses caused by aging affect people and can cause them to lose their ability to perform daily routine functions. In order to help people recover or improve their dysfunctional activities and quality of life after accidents or strokes, assistive devices like exoskeletons and orthoses are developed. Control strategies for control of exoskeletons are developed with the desired intention of improving the quality of treatment. Amongst recent control strategies used for rehabilitation robots, active disturbance rejection control (ADRC) strategy is a systematic way out from a robust control paradox with possibilities and promises. In this modern era, we always try to find the solution in order to have minimum resources and maximum output, and in robotics-control, to approach the same condition observer-based control strategies is an added advantage where it uses a state estimation method which reduces the requirement of sensors that is used for measuring every state. This paper introduces improved active disturbance rejection control (I-ADRC) controllers as a combination of linear extended state observer (LESO), tracking differentiator (TD), and nonlinear state error feedback (NLSEF). The proposed controllers were evaluated through simulation by investigating the sagittal plane gait trajectory tracking performance of two degrees of freedom, Lower Limb Robotic Rehabilitation Exoskeleton (LLRRE). This multiple input multiple output (MIMO) LLRRE has two joints, one at the hip and other at the knee. In the simulation study, the proposed controllers show reduced trajectory tracking error, elimination of random, constant, and harmonic disturbances, robustness against parameter variations, and under the influence of noise, with improvement in performance indices, indicates its enhanced tracking performance. These promising simulation results would be validated experimentally in the next phase of research.

## 1. Introduction

### 1.1. Motivation and Background

Classically, the physiotherapist’s job is to assist the patient in performing various rehabilitative tasks, and help them to recover or improve natural strength and movements through a set of objective oriented exercises. Re-iteration of such tasks for a long time results in fatigue of vitality, and it tires both the patient and the therapist [1,2]. In addition, it is difficult for the patient to visit physiotherapy centers for rehabilitation frequently due to physical conditions as well as traveling may affect the safety of the patient. There is no quantitative analysis for the patient’s recuperation that can be acquired subsequent to the performance of rehabilitative exercises. Robotic rehabilitation devices can be ameliorated in such cases to overcome the difficulties of therapeutic training [3,4] as they are designed for the purpose of objective oriented tasking and can frequently work on the same trajectory for the duration of time depending on the patient’s comfort. The performance tracking after the training through these devices leads to reinforcing the recovery of the patient, where, validation can be established by improvement comparison on the measurement result.

The design of control using a state feedback controller requires the availability of all state variables, but this condition may not always be true; in some cases, due to faulty sensors, these states are unavailable or immeasurable. Hence, observer based technique is utilized in this paper to reconstruct such state variables. Again, utilization of sensors for measuring all parameters is a costly process and adds extra hardware [5].

### 1.2. Related Research

Different control schemes for the improvement in the area of Lower Limb Robotic Rehabilitation Exoskeleton (LLRRE) for human assistance have been developed. Proportional-Derivative (PD) based control shows good performance in the absence of disturbance [6], but usually suffers when the disturbance occurs in the system [7]. Particle swarm optimization (PSO) based active force rejection control is introduced in [8] for rejecting the disturbance in gait trajectory tracking requires evaluation of a large number of parameters. Computed-torque control (CTC) [9,10] depends on the exact model of the system and may require additional control to compensate for modeling errors. Intelligent control methods [11] require great effort in rule formulation and inference testing. Sensitivity amplification needs an accurate inverse dynamic model and suffers from the introduction of disturbance [12,13]. Radial Basis Functions (RBF) neural network used to compensate for the disturbance but results in large computing costs [14]. Robust control (RC) methods are one of the options in such scenarios, but RC techniques are conservative and consider the worst-case approaches at the cost of relinquishing the transient response. The sliding mode control (SMC) technique can restrain against the uncertainties and parameter fluctuations, but faces chattering due to discontinuous switching [15]. To overcome such modern control difficulties, the active disturbance rejection control (ADRC) method is proposed.

The control theory requires a great effort on system identification, i.e., the mathematical model of the system and obtaining a perfect one is an ideal case. The ADRC controller was firstly proposed by Han [16] and had many advantages. The evolution and rapid use of ADRC in industries in the last three decades prove its popularity in motion control [17,18,19,20], flight control [21,22], and process control [23,24,25,26,27,28,29,30,31,32] applications and in many fields [33]. The architecture of ADRC is designed to achieve the best performance by actively eliminating the internal and outside uncertainties as a entire disturbance [34]. Its significance is being found out in industries to be a replacement for proportional–integral–derivative (PID). ADRC inherits from PID, but it has improved characteristics. It is based on error-driven rather than model-based control law [35] and does not necessarily depend on full information of the model or system dynamics, i.e., eliminates the necessity of the exact model of the system [36]. ADRC is recognized as a model-free controller, It only requires the order of the system and the approximate value of system parameters [37]. In the category of lower limb rehabilitation, robotic devices, orthoses, exoskeleton, and prosthesis are developed to assist users mainly for gait rehabilitation and other exercises like sitting, standing, etc. Orthoses and exoskeleton have similar functionality [38]. In recent years, due to popularity and effectiveness, ADRC is used for various robotic rehabilitation devices for tracking applications. A linear extended state observer (LESO) based ADRC has applied on the lower limb exoskeleton for the hip and knee joints in [39] where clinical gait data is used as a reference. Results are compared for PID and ADRC, for the hip and knee trajectories based on error comparison the results show a better performance of ADRC over PID. To keep track of active ankle-foot orthosis (AAFO) [40], a framework similar to [39] is used in which the authors modified the ADRC with the inclusion of Control Lyapunov Function (CLF) instead of PD controller, with Sontag’s formula. Stability is checked by input to state (ISS) framework where modification and experiments prove ARDC’s effectiveness. In another work [41], ADRC deals with nonlinearities like pressure fluctuation and friction during the control of exoskeleton, a new function is introduced to avoid shaking at inflation point during non-linear state error feedback (NLSEF). A sinusoidal tracking for exoskeleton joint output is compared with ADRC, and for disturbance rejection, it is compared with PID, where ADRC with NLSEF shows better results than ADRC.

There are several control strategies for rehabilitation, such as position tracking, force and impedance control, biosignals based control and adaptive control, etc [42,43,44,45,46,47]. Position tracking is one of the basic control strategies for robotic rehabilitation devices in which repeatability and position accuracy of motion are improved by the help of the controller for the patient’s recovery [3,39,48,49,50].

### 1.3. Contribution and Paper Structure

In this paper, more focus is given to position tracking control of predefined sagittal plane gait trajectory. This paper centers around the design of controllers for lower limb robotic rehabilitation exoskeleton (LLRRE) for sagittal plane gait trajectory tracking control based on ADRC combinations. A nonlinear dynamic, multiple-input multiple-output (MIMO) LLRRE with two joints, one at the hip and other at the knee obtained via Euler–Lagrange method, is presented. The proposed controller is a combination of three units, linear extended state observer (LESO), Tracking differentiator (TD), and Nonlinear state error feedback (NLSEF). LESO is used to estimate the states of the system, eliminates disturbance, and control the system by linear or nonlinear gains. NLSEF uses nonlinear gains to take care of the overshoot and speed of response. TD operates on a transient profile of nonlinear input signals by differentiating it, which results in the gradual increasing output instead of sudden changes. The proposed work is verified by performing numerous simulations and on the basis of various performance indices. This new combination of three units results in improved gait trajectory tracking and disturbance rejection performance.

In this paper, Section 2 gives the modeling of the exoskeleton understudy, and Section 3 presents the theory of the proposed control strategy for LLRRE. Section 4 gives the design of the ADRC for the trajectory tracking controller. Section 5 gives the stability of the proposed control method. The results of simulations are highlighted and discussed in Section 6 and Section 7. Finally, the conclusion is given in Section 8.

## 2. Modeling of Lower Limb Robotic Rehabilitation Exoskeleton

**Figure 1:**
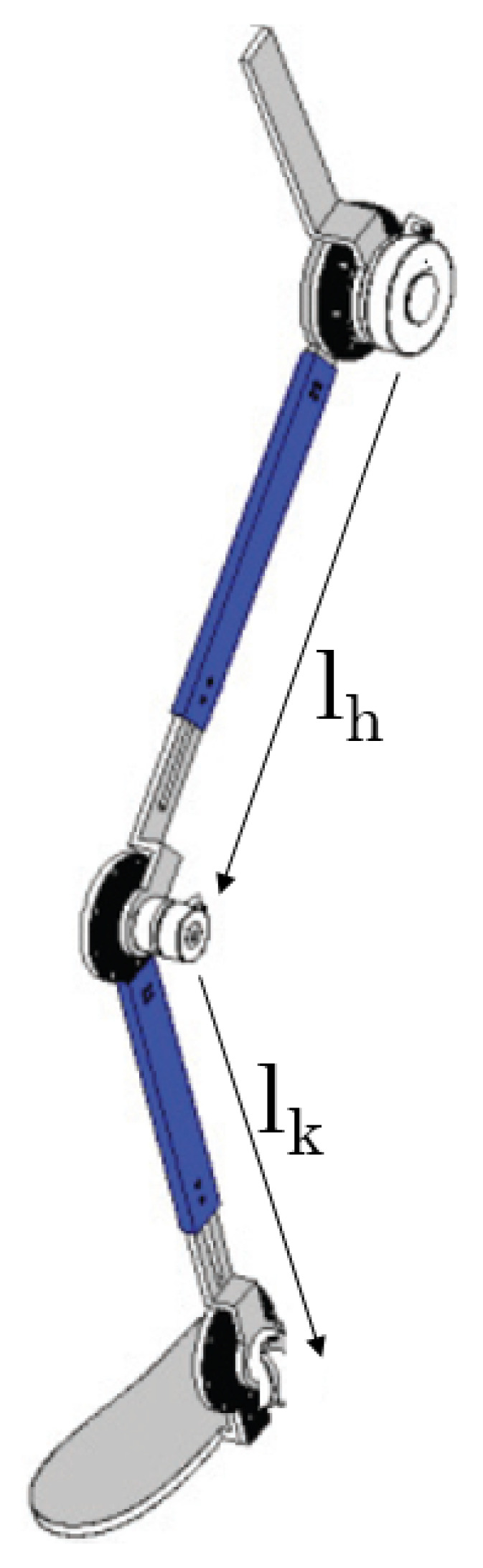
Lower Limb Robotic Rehabilitation Exoskeleton.

An exoskeleton design must be biology-inspired to provide multi-functionality and adaptability to users, a similar approach called clinical gait data analysis is used here for lower limb exoskeleton modeling and design. The model consists of the hip and knee joint movements, which are provided with the help of two electric motors embedded in the structure. The model used in this paper is based on [39]. Figure 1 gives the structure of the exoskeleton and the parameters of exoskeleton are listed in Table 1.

The Euler–Lagrange method is used for the mathematical modeling, the swing of the leg is given by

(1) $$\begin{array}{c} \begin{array}{c} M\left(q\right)\overset{..}{q}+C\left(q,\dot{q}\right)\dot{q}+G\left(q\right)+D=T \end{array} \end{array}$$

where,

$M\;\left(q\right)\in R^{n\times n}$ is the symmetric definite inertial matrix.

$C\;\left(q,\dot{q}\right)\in R^{n\times n}$ is the Coriolis and centrifugal force matrix.

$G\;\left(q\right)\in R^{nx1}$ is the gravitational force matrix.

$T\in R^{n\times 1}$ is the control input vector.

$D\in R^{n\times 1}$ denotes un-modelities and exogenous disturbance.

Properties for dynamic modeling in Equation (1), are as follows:

1. Matrix M(q) is symmetric and positive definite.
2. Matrix $\dot{M}\left(\dot{q}\right)-2C\left(q,\dot{q}\right)$ is a skew-symmetric matrix if $\forall \varepsilon \in R^{n},\varepsilon^{T}\left(\dot{M}\left(\dot{q}\right)-2C\left(q,\dot{q}\right)\right)\varepsilon =0.$
3. There exist finite scalars $\delta_{i}>0,\;i=1,..,4$ such that $∥M\left(q\right)∥\leq \delta_{1},∥C\left(q,\dot{q}\right)∥\leq \delta_{2},∥G\left(q\right)∥\leq \delta_{3}\;and\;∥D∥\leq \delta_{4}$ which means all items in dynamic model are bounded.

$q={\left[q_{h}\;q_{k}\right]}^{T}$, where $q_{h}$ and $q_{k}$ represent angular position for the hip and knee joints. $T={\left[\tau_{h}\;\tau_{k}\right]}^{T}$, where $\tau_{h}$ and $\tau_{k}$ represent driving torque for the hip and knee joints.

The equations of matrices are as follows:

(2) $$\begin{array}{c} \begin{array}{c} \begin{array}{c} \begin{array}{c} M\left(q\right)=\left[\begin{array}{cc} m_{11} & m_{12}\\ m_{21} & m_{22} \end{array}\right] \end{array}\\ m_{11}=\frac{1}{3}m_{h}l_{h}^{2}+m_{k}l_{h}^{2}+\frac{1}{4}m_{k}l_{k}^{2}+m_{k}l_{h}l_{k}\cos\left(q_{k}\right)\\ m_{12}=-\frac{1}{4}m_{k}l_{k}^{2}-\frac{1}{2}m_{k}l_{h}l_{k}\cos\left(q_{k}\right)\\ m_{21}=-\frac{1}{4}m_{k}l_{k}^{2}-\frac{1}{2}m_{k}l_{h}l_{k}\cos\left(q_{k}\right)\\ m_{22}=\frac{1}{3}m_{k}l_{k}^{2} \end{array} \end{array} \end{array}$$

(3) $$\begin{array}{c} \begin{array}{c} \begin{array}{c} C\left(q,\dot{q}\right)=\left[\begin{array}{cc} c_{11} & c_{12}\\ c_{21} & c_{22} \end{array}\right]\\ c_{11}=-m_{k}l_{h}l_{k}{\dot{q}}_{k}\sin\left(q_{k}\right)\\ c_{12}=\frac{1}{2}m_{k}l_{h}l_{k}{\dot{q}}_{h}\sin\left(q_{k}\right)\\ c_{21}=\frac{1}{2}m_{k}l_{h}l_{k}{\dot{q}}_{h}\sin\left(q_{k}\right)+\frac{1}{2}m_{k}l_{h}l_{k}{\dot{q}}_{h}\sin\left(q_{k}\right)\\ c_{22}=\frac{1}{2}m_{k}l_{h}l_{k}{\dot{q}}_{h}\sin\left(q_{k}\right) \end{array} \end{array} \end{array}$$

$\dot{q_{h}}$ and $\dot{q_{k}}$ represent velocities of the hip and knee joints. G(q) is expressed as:

(4) $$\begin{array}{c} \begin{array}{c} \begin{array}{c} \begin{array}{c} \begin{array}{c} G\left(q\right)=\left[\begin{array}{c} g_{1}\\ g_{2} \end{array}\right] \end{array}\\ g_{1}=-\frac{1}{2}m_{h}l_{h}g\sin\left(q_{h}\right)-m_{k}l_{h}g\sin\left(q_{h}\right)-\frac{1}{2}m_{k}l_{k}g\sin\left(q_{h}-q_{k}\right)\\ g_{2}=\frac{1}{2}m_{k}l_{k}g\sin\left(q_{h}-q_{k}\right) \end{array} \end{array} \end{array} \end{array}$$

for the model of robotic exoskeleton, the error for trajectory tracking is defined as

(5) $$\begin{array}{c} \begin{array}{c} e\;=\;q_{d}-\;q \end{array} \end{array}$$

where, e is the tracking error. $q_{d}\;$ and q are desired and actual trajectories, respectively.

In the starting phase of rehabilitation and in passive mode, the exoskeleton allows the patient to move in the well-known predefined trajectory to initialize the joint movements. The objective of the rehabilitation exoskeleton in this paper is to replicate the exact gait pattern with high precision under the influence of noise and disturbances. The trajectories for the hip and knee joints are obtained by using fitting expression using clinical gait analysis data [51]. The period of the cyclical gait is 2 s and the fitting expression with respect to time is obtained as follows and considering the gait cycle starts at stance phase initially and then repeats.

(6) $$\begin{array}{c} \begin{array}{c} \left\{\begin{array}{c} \begin{array}{c} q_{h,d}\left(t\right)=c_{0}.\cos\left(0.d.t\right)+c_{1}.\cos\left(1.d.t\right)+f_{1}.\sin\left(1.d.t\right)\\ \;\;\;\;\;\;\;\;\;\;\;\;\;\;\;+c_{2}.\cos\left(2.d.t\right)+f_{2}.\sin\left(2.d.t\right)\\ \;\;\;\;\;\;\;\;\;\;\;\;\;\;\;+c_{3}.\cos\left(3.d.t\right)+f_{3}.\sin\left(3.d.t\right)-29.1{}^{\circ}\\ q_{k,d}\left(t\right)=c_{4}.\cos\left(0.d_{1}.t\right)+c_{5}.\cos\left(1.d_{1}.t\right)+f_{4}.\sin\left(1.d_{1}.t\right)\\ \;\;\;\;\;\;\;\;\;\;\;\;\;\;\;\;\;+c_{6}.\cos\left(2.d_{1}.t\right)+f_{5}.\sin\left(2.d_{1}.t\right)\\ \;\;\;\;\;\;\;\;\;\;\;\;\;\;\;\;\;\;+c_{7}.\cos\left(3.d_{1}.t\right)+f_{6}.\sin\left(3.d_{1}.t\right)-26.127{}^{\circ} \end{array} \end{array}\right. \end{array} \end{array}$$

**Figure 2:**
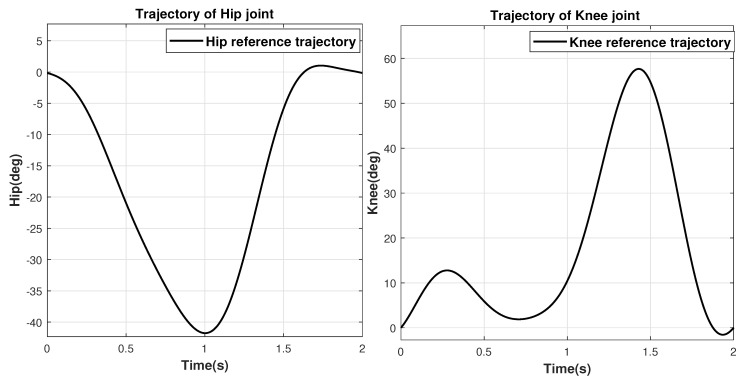
Reference trajectories of the hip and knee joints.

The values of coefficients are shown in Table 2 and Figure 2 shows the respective trajectories for the hip and knee joints.

In this paper, the reference trajectories i.e., predefined gait trajectories representing desired angular positions of the hip and knee joints, are obtained using Equation (6) for the analysis of the proposed algorithm. The gait cycle in Equation (6) starts initially at stance phase and continues periodically every 2 s as shown in Figure 2.

## 3. Active Disturbance Rejection Control for Lower Limb Robotic Rehabilitation Exoskeleton

**Figure 3:**
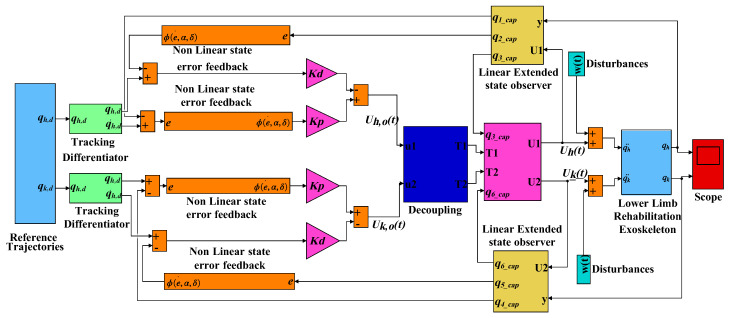
Topology of proposed active disturbance rejection control (ADRC) for lower limb robotic rehabilitation exoskeleton (LLRRE).

Figure 3 shows the block diagram of the proposed ADRC applied to the LLRRE. In this paper, the MIMO system is first converted to a single input single output (SISO) by decoupling, for the hip and the knee joint, and then the proposed controller is applied.

The proposed ADRC method as a combination of LESO, TD, and NLSEF eliminates the un-modeled dynamics and uncertainties of the system, improves the dynamic response of the system, and reduces overshoot.

Where, $q_{h,d}\left(t\right)$ and $q_{k,d}\left(t\right)$ are the respective reference trajectories for the hip and knee joints. ${\hat{q}}_{1}\left(t\right),{\hat{q}}_{2}\left(t\right),{\hat{q}}_{4}\left(t\right)$, and ${\hat{q}}_{5}\left(t\right)$ are the estimated and ${\hat{q}}_{3}\left(t\right),{\hat{q}}_{6}\left(t\right)$ are extended states of the LESO for called LESO for the hip joint and LESO for the knee joint, respectively. $U_{h,0}\left(t\right)$ and $U_{k,0}\left(t\right)$ are the outputs of the NLSEF. $U_{h}\left(t\right)$ and $U_{k}\left(t\right)$ are the outputs of the improved ADRC. w(t) is the exogenous disturbance. $q_{h}\left(t\right)\;and\;q_{k}\left(t\right)\;$ denotes the actual angular position respectively for hip and the knee joints.

### 3.1. Linear Extended State Observer (LESO) 

ADRC is an observer-based control strategy that makes use of an LESO [52,53]. LESO is the core in the architecture of ADRC, which makes use of available knowledge for interpretation of the states, online estimates states, and eliminates the caveats like model parameters, exogenous signals, and uncertainties as a total disturbance. The general design of LESO for the SISO system, followed by second-order LESO, is presented in this subsection.

The generalized nth order system (SISO) is presented as follows:

(7) $$\begin{array}{c} \begin{array}{c} y^{\left(n\right)}\left(t\right)=f\left(y\left(t\right),\dot{y}\left(t\right),\;.\;.\;.\;,\;y^{\left(n-1\right)}\left(t\right),w\left(t\right),t\right)+bu\left(t\right) \end{array} \end{array}$$

where, *w*(*t*) is the exogenous disturbance, *u*(*t*), input, *y*(*t*), output, *b* is the system parameter $f(y\left(t\right),\dot{y}\left(t\right),\;.\;.\;.\;,y^{\left(n-1\right)}\left(t\right),w\left(t\right),t)$ comprising exogenous disturbance and internal modeling uncertainties called as an entire disturbance.

Let, $q_{1\;}=y,\;q_{2}=\dot{y,}\;q_{3}=\overset{..}{y},\;.\;.\;.\;,q_{n}=y^{\left(n-1\right)}$, and putting in Equation (7) gives

(8) $$\begin{array}{c} \begin{array}{c} \begin{array}{c} \left\{\begin{array}{c} \begin{array}{c} {\dot{q}}_{1}=q_{2},\\ {\dot{q}}_{2}=q_{3},\\ .\\ .\\ {\dot{q}}_{n-1}=q_{n},\;\\ {\dot{q}}_{n}=f\left(q_{1},q_{2},\;.\;.\;.\;.\;,q_{n},w\left(t\right),t\right)+bu,\\ y=q_{1} \end{array} \end{array}\right. \end{array} \end{array} \end{array}$$

The variable $q_{n+1}$ in Equation (9) is augmented and introduced in the architecture of an (LESO) in Equation (8).

(9) $$\begin{array}{c} \begin{array}{c} q_{n+1}=f\left(q_{1},q_{2},\;.\;.\;.\;,q_{n},w\left(t\right),t\right) \end{array} \end{array}$$

For linearization of the system in Equation (8). The combination of Equation (8) with Equation (9) gives the extended-state equation, as follows

(10) $$\begin{array}{c} \begin{array}{c} \begin{array}{c} \left\{\begin{array}{c} \begin{array}{c} \begin{array}{c} {\dot{q}}_{1}=q_{2},\\ {\dot{q}}_{2}=q_{3},\\ .\\ .\\ {\dot{q}}_{n-1}=q_{n},\;\\ {\dot{q}}_{n}=q_{n+1}+bu,\;\\ {\dot{q}}_{n+1}=h\left(t\right), \end{array}\\ \;y=\;q_{1} \end{array} \end{array}\right. \end{array} \end{array} \end{array}$$

where, $h\left(t\right)=f(q_{1},q_{2},...,q_{n},w\left(t\right),t).$

For estimation of extended states, a LESO is generally designed as

(11) $$\begin{array}{c} \begin{array}{c} \left\{\begin{array}{c} \begin{array}{c} {\dot{\hat{q}}}_{1}\;=\;{\hat{q}}_{2}+\;\beta_{1}\left(q_{1}-{\hat{q}}_{1}\;\right),\;\\ {\dot{\hat{q}}}_{2}\;=\;{\hat{q}}_{3}+\beta_{2}\left(q_{1}-{\hat{q}}_{1}\;\right),\\ .\\ .\\ \;{\dot{\hat{q}}}_{n}=\;{\hat{q}}_{n+1\;}+\beta_{n}\left(q_{1}-{\hat{q}}_{1}\;\right)+bu,\\ \;{\dot{\hat{q}}}_{n+1}=\beta_{n+1}\left(q_{1}-{\hat{q}}_{1}\;\right), \end{array} \end{array}\right. \end{array} \end{array}$$

where, ${\hat{q}}_{1},{\hat{q}}_{2},...,{\hat{q}}_{n}$, and ${\hat{q}}_{n+1}$ are estimates of states $q_{1},q_{2},...,q_{n}$, and $q_{n+1},$ respectively, and $\beta_{1},\beta_{2},...,\beta_{n+1}$ are the observer gains to be designed.

$e_{i}=q_{i}-{\hat{q}}_{i}\;\left(i=1,2,...,n+1\right)$ denotes the error for state estimation.

For the second-order system, the LESO can be modeled as

(12) $$\begin{array}{c} \begin{array}{c} \left\{\begin{array}{c} \begin{array}{c} {\dot{\hat{q}}}_{1}={\hat{q}}_{2}+\beta_{1}\left(q_{1}-{\hat{q}}_{1}\;\right),\;\\ {\dot{\hat{q}}}_{2}={\hat{q}}_{3}+\beta_{2}\left(q_{1}-{\hat{q}}_{1}\;\right)+bu,\\ \;{\dot{\hat{q}}}_{3}=\beta_{3}\left(q_{1}-{\hat{q}}_{1}\;\right)\; \end{array} \end{array}\right. \end{array} \end{array}$$

Equation (12) can be written in state-space form as

(13) $$\begin{array}{c} \begin{array}{c} \left\{\begin{array}{c} \overset{\dot{\wedge }}{Q}=A\overset{\wedge }{Q}+Bu+\beta e\\ y=C\overset{\wedge }{Q} \end{array}\right. \end{array} \end{array}$$

where,

(14) $$\begin{array}{c} \begin{array}{c} A=\left[\begin{array}{ccc} 0 & 1 & 0\\ 0 & 0 & 1\\ 0 & 0 & 0 \end{array}\right],B=\left[\begin{array}{c} 0\\ b\\ 0 \end{array}\right],\beta =\left[\begin{array}{c} \beta_{1}\\ \beta_{2}\\ \beta_{3} \end{array}\right],C=\left[\begin{array}{c} 1\\ 0\\ 0 \end{array}\right] \end{array} \end{array}$$

$\beta ={\left[\begin{array}{ccc} \beta_{1}, & \beta_{2}, & \beta_{3} \end{array}\right]}^{T}$ represents observer gain vector. For simplifying tuning, all the observer poles are placed at $-\omega_{o}$. For determination of observer gains, the following characteristic equation is used [54].

(15) $$\begin{array}{c} \begin{array}{c} \lambda_{0}\;\left(s\right)=s^{3}+\beta_{1}s^{2}+\beta_{2}s+\beta_{3}={\left(s+w_{0}\right)}^{3} \end{array} \end{array}$$

where, $w_{o}$ represents bandwidth of observer. For the above characteristic equation, values of gain vector β are expressed by $\beta_{1}=3w_{0},\beta_{2}=3w_{0}^{2},\beta_{3}=w_{0}^{3}$.

The ADRC control law is defined by $u=\frac{u_{0}-\overset{\wedge }{f}}{b}$, where *b* represents system parameter, depends on the system dynamics, $u_{0}=K_{p}\left(q_{d}-\overset{\wedge }{q_{1}}\right)+K_{d}\left({\dot{q}}_{d}-\overset{\wedge }{q_{2}}\right)+{\overset{..}{q}}_{d}$ with well-designed ESO, the last term in left-hand side $\overset{..}{q}$ is very small and the rest of the terms constitute a proportional derivative controller [55]. $k_{p}=w_{c}^{2}$ and $K_{d}=2w_{c}$ are the selected controller gains [56].

### 3.2. Tracking Differentiator (TD)

The TD is generally implemented to avoid overshoot and optimize the system response [57]. It operates on a transient profile of input signals, differentiated it to avoid abrupt change, which results in the gradual increasing output instead of sudden changes. In this paper TD developed by Zhigao Liu [58] shown in Equation (16), which is easy to implement and superior to the classical nonlinear tracking differentiator, is used to improve trajectory tracking performance.

(16) $$\begin{array}{c} \begin{array}{c} \begin{array}{c} {\dot{x}}_{1}=x_{2}\\ {\dot{x}}_{2}=-R^{2}\;\left(a_{1}\left[x_{1}-\nu \left(t\right)\right]-b_{1}\;\frac{x_{2}\left(t\right)}{R}-b_{2}\frac{x_{2}{\left(t\right)}^{n}}{R^{n}}\right) \end{array} \end{array} \end{array}$$

*a${}_{1}$ > 0, b${}_{1}$ > 0, b${}_{2}$ > 0, n > 0, n* is odd.

Here, *x*${}_{1\;}$is the desired trajectory and *x*${}_{2\;}$is its derivative. The selection of *R* depends on application and selected appropriately to adjust the pace of the transient profile. Then, *x*${}_{2\;}$is denoted as the “tracking differentiator” of *v(t).*

### 3.3. Non-Linear State Error Feedback (NLSEF)

In this paper a NLSEF based on Equation (17) given by J. Han [16] and Wu Qing Xu [41] in Equation (18) is used. A non-linear state error feedback function *fal*(*.*) is represented by the form

(17) $$\begin{array}{c} \begin{array}{c} fal\left(e,\alpha ,\delta \right)=\left\{\begin{array}{c} \frac{e}{\delta^{1-\alpha }}\;\;\;\;\;\;\;\;\;\;\;\;\;\;\;\left|e\right|<\delta \\ {\left|e\right|}^{\alpha }.\;sign\left(e\right),\;\;\;\left|e\right|>\delta \end{array}\right. \end{array} \end{array}$$

δ and α are the tuning parameter for the exponential function. There exists linear regions for the *fal*(*.*) and is not a smooth curve which lead to flutter the controller, a new function is used which has a smooth curve is presented in Equation (18).

(18) $$\begin{array}{c} \begin{array}{c} \left\{\begin{array}{c} \begin{array}{c} \begin{array}{c} \begin{array}{c} \phi (e,\alpha ,\delta )=newfal(e,\alpha ,\delta )=\\ \left(\alpha -1\right)\delta^{\alpha -3}e^{3}-\left(\alpha -1\right)\delta^{\alpha -2}e^{2}sign\left(e\right)\\ +\delta^{\alpha -1}e, \end{array} \end{array}\;\;\left|e\right|<\delta \end{array}\\ {\;\;\;\;\;\;|e|}^{\alpha }sign\left(e\right),\;\;\;\;\;\;\;\;\;\;\;\;\;\;\;\;\;\;\;\;\;\;\;\;\;\;\;\;\;\;\;\;\;\;\;\;\;\;\;\;\;\;\;\;\;\;\left|e\right|>\delta \end{array}\right. \end{array} \end{array}$$

The nonlinear control feedback law non-linearly combines the error between state error feedback and reference, and estimated state.

## 4. Design of ADRC for Trajectory Tracking Controller

The mathematical model in Equation (1) can be expressed as follows:

(19) $$\begin{array}{c} \begin{array}{c} \left\{\begin{array}{c} m_{11}\overset{..}{q_{h}}+m_{12}\overset{..}{q_{k}}+c_{11}\dot{q_{h}}+c_{12}\dot{q_{k}}+g_{1}+D_{1}=\tau_{1}\\ m_{21}\overset{..}{q_{h}}+m_{22}\overset{..}{q_{k}}+c_{21}\dot{q_{h}}+c_{22}\dot{q_{k}}+g_{2}+D_{2}=\tau_{2} \end{array}\right. \end{array} \end{array}$$

where, $D={\left[\begin{array}{cc} D_{1}, & D_{2} \end{array}\right]}^{T}$ and $T={\left[\begin{array}{cc} \tau_{1}, & \tau_{2} \end{array}\right]}^{T}$, $D_{1}$ and $D_{2}$ are exogenous disturbances and un-modeled dynamics, $\tau_{1}$ and $\tau_{2}$ are torques of the hip and knee joints.

Equation (19) in state space is given as:

(20) $$\begin{array}{c} \begin{array}{c} \left\{\begin{array}{c} \overset{..}{q_{h}}=\frac{1}{\left(m_{11}m_{22}-m_{21}m_{12}\right)}.\left(m_{22}\tau_{1}-m_{12}\tau_{2}-F_{1}\right)\\ \overset{..}{q_{k}}=\frac{1}{\left(m_{12}m_{21}-m_{11}m_{22}\right)}.\left(m_{21}\tau_{1}-m_{11}\tau_{2}-F_{2}\right) \end{array}\right. \end{array} \end{array}$$

where,

(21) $$\begin{array}{c} \begin{array}{c} \left\{\begin{array}{c} \begin{array}{c} F_{1}=\left(m_{22}c_{11}-m_{12}c_{21}\right){\dot{q}}_{h}+\left(m_{22}c_{12}-m_{12}c_{22}\right){\dot{q}}_{k}\\ +m_{22}g_{1}-m_{12}g_{2}+m_{22}D_{1}-m_{12}D_{2}\\ F_{2}=\left(-m_{21}c_{11}-m_{11}c_{21}\right){\dot{q}}_{h}-\left(m_{21}c_{12}-m_{11}c_{22}\right){\dot{q}}_{k}\\ -m_{21}g_{1}+m_{11}g_{2}-m_{22}D_{1}+m_{12}D_{2} \end{array} \end{array}\right. \end{array} \end{array}$$

Equation (21) can be written as:

(22) $$\begin{array}{c} \begin{array}{c} \left\{\begin{array}{c} \overset{..}{q_{h}}=C_{f1}.\left(m_{22}\tau_{1}-m_{12}\tau_{2}\right)+f_{1}\\ \overset{..}{q_{k}}=-C_{f1}.\left(m_{21}\tau_{1}-m_{11}\tau_{2}\right)+f_{2} \end{array}\right. \end{array} \end{array}$$

where, $C_{f1}=\frac{1}{\left(m_{11}m_{22}-m_{21}m_{12}\right)},\;f_{1}=-F_{1}C_{f1},f_{2}=F_{2}C_{f1}$.

The system in Equation (22) is decoupled by matrix *D*.

(23) $$\begin{array}{c} \begin{array}{c} D=\left[\begin{array}{cc} Cf_{1}m_{22} & 0\\ 0 & Cf_{1}m11 \end{array}\right] \end{array} \end{array}$$

The system Equation (22) can be expressed as

(24) $$\begin{array}{c} \begin{array}{c} \overset{..}{q}\;=\;f\;+\;U \end{array} \end{array}$$

here, $\overset{..}{q}\;=\;{\left[{\overset{..}{q}}_{h},\;{\overset{..}{q}}_{k}\;\right]}^{T}\;,\;f\;=\;{\left[\;f_{1},\;f_{2}\right]}^{T},U\;=\;{\left[U_{1},\;U_{2}\right]}^{T}\;=\;D{\left[\tau_{1},\tau_{2}\right]}^{T}\;$if *U* is known, the control of input $T={\left[\begin{array}{cc} \tau_{1}, & \tau_{2} \end{array}\right]}^{T}$ can be obtained as $\;D_{inv}U$ after the system is decoupled, ADRC can be split into two independent equations for the hip and knee joint.

(25) $$\begin{array}{c} \begin{array}{c} \begin{array}{c} \overset{..}{q_{h}}\;=\;f_{1\;}+\;D_{\mathrm{inv}}\left(1,1\right).U_{1}\\ \overset{..}{q_{k}}\;=\;f_{2}\;+\;D_{\mathrm{inv}}\left(2,2\right).U_{2} \end{array} \end{array} \end{array}$$

Based on Equation (12) and Equation (25) the LESO is designed separately for each joint. Two LESOs are

(26) $$\begin{array}{c} \begin{array}{c} \left\{\begin{array}{c} \begin{array}{c} {\dot{\hat{q}}}_{1}={\hat{q}}_{2}+\beta_{1}\left(q_{1}-{\hat{q}}_{1}\;\right),\;\\ {\dot{\hat{q}}}_{2}={\hat{q}}_{3}+\beta_{2}\left(q_{1}-{\hat{q}}_{1}\;\right)+D_{\mathrm{inv}}\left(1,1\right).U_{1},\\ \;{\dot{\hat{q}}}_{3}=\beta_{3}\left(q_{1}-{\hat{q}}_{1}\;\right)\;\\ \; \end{array}\\ \begin{array}{c} {\dot{\hat{q}}}_{4}={\hat{q}}_{5}+\beta_{4}\left(q_{1}-{\hat{q}}_{1}\;\right),\;\\ {\dot{\hat{q}}}_{5}={\hat{q}}_{6}+\beta_{5}\left(q_{1}-{\hat{q}}_{1}\;\right)+D_{\mathrm{inv}}\left(2,2\right).U_{2},\\ \;{\dot{\hat{q}}}_{6}=\beta_{6}\left(q_{1}-{\hat{q}}_{1}\;\right)\; \end{array} \end{array}\right. \end{array} \end{array}$$

where,$\;\beta_{one}={\left[\beta_{1},\;\beta_{2},\;\beta_{3}\right]}^{T}$ and $\beta_{two}={\left[\beta_{4},\;\beta_{5},\;\beta_{6}\right]}^{T}$ are the observer gain matrices. In this paper the bandwidth $\omega_{0}$ for all the LESO observers are of same value. The bandwidth of feedback controller is defined as$\;w_{c}=\frac{1}{3}w_{0}$. $K_{p}=\left[\begin{array}{cc} w_{c}^{2} & 0\\ 0 & w_{c}^{2} \end{array}\right],\;K_{d}=\left[\begin{array}{cc} 2w_{c} & 0\\ 0 & 2w_{c} \end{array}\right]\;$are the gain matrices of controller.

The feedback control law for LESO can be written as $\;U_{0}={\left[U_{1},U_{2}\right]}^{T\;}=K_{p}e+K_{d}\dot{e}$, e, and $\dot{e}$ are the state estimation errors for the position and velocity respectively for the hip and knee joints. $q_{d}=\left[\begin{array}{cc} q_{h,d} & q_{k,d} \end{array}\right]$ as reference trajectory for the hip and knee joints and $e={\left[\begin{array}{cc} e_{1} & e_{2} \end{array}\right]}^{T}={\left[\begin{array}{cc} \left(q_{h,d}-\overset{\wedge }{q_{1}}\right) & \left(q_{k,d}-\overset{\wedge }{q_{4}}\right) \end{array}\right]}^{T},\;\dot{e}={\left[\begin{array}{cc} {\dot{e}}_{1} & {\dot{e}}_{2} \end{array}\right]}^{T}=\left[\begin{array}{cc} \left({\dot{q}}_{h,d}-\overset{\wedge }{q_{2}}\right) & \left({\dot{q}}_{k,d}-\overset{\wedge }{q_{5}}\right) \end{array}\right]$.

The ADRC strategy for standard second order integrator $y=U_{0}\;$can be expressed as $U={\left[\tau_{1},\tau_{2}\right]}^{T}=D_{inv}.\left(K_{p}e+K_{d}\dot{e}-\overset{\wedge }{f}\right)$. $\overset{\wedge }{f}={\left[\;{\hat{f}}_{1},\;{\hat{f}}_{2}\right]}^{T}$ for the estimated disturbances.

The control law for ADRC designed from Equations (12), (16), (18) and (25) is given in Table 3.

The system in Equation (7) finally becomes Equation (27) for well-designed control law as shown in Equation (24)

(27) $$\begin{array}{c} \begin{array}{c} \overset{..}{q}=f+D_{inv}\left(\left(k_{p}\phi \left(e,\delta ,\alpha \right)+k_{d}\dot{e}\right)+{\overset{..}{q}}_{d}-\hat{f}\right) \end{array} \end{array}$$

$\overset{..}{q}={\left[{\dot{q}}_{2},\;{\dot{q}}_{4}\right]}^{T}$. In the control laws $q_{d}={\left[\begin{array}{cc} q_{h,d} & q_{k,d} \end{array}\right]}^{T}$ is the desired gait trajectory of the joints of exoskeleton. ${\overset{..}{q}}_{d}={\left[\begin{array}{cc} {\overset{..}{q}}_{h,d} & {\overset{..}{q}}_{k,d} \end{array}\right]}^{T}$ is negligible, $f={\left[\begin{array}{cc} q_{3} & q_{6} \end{array}\right]}^{T}$ whereas $\overset{\wedge }{q_{1}},\;\overset{\wedge }{q_{2}},\;\overset{\wedge }{q_{4}},\;\overset{\wedge }{q_{5}}$ are the estimated states for $\overset{}{q_{1}},\;\overset{}{q_{2}},\;\overset{}{q_{4}},\;\overset{}{q_{5}}$, and $\hat{f}={\left[\overset{\wedge }{q_{3}},\;\overset{\wedge }{q_{6}}\;\right]}^{T}$ represents the extended state which eliminates the disturbances and uncertainties that occur in the system.

## 5. Stability Analysis

Assuming $f(q_{1},q_{2},....q_{n},u,\omega \left(t\right),t)$ is globally Lipschitz with respect to *q*, there exists a constant $w_{0}>0$, $w_{c}>0$, such that the closed loop system Equation (27) is asymptotically stable.

**Proof.**  From Table 3 one has

(28) $$\begin{array}{c} \begin{array}{c} U=D_{inv}\left(\left(k_{p}\phi \left(e,\delta ,\alpha \right)+k_{d}\dot{e}\right)+{\overset{..}{q}}_{d}-\hat{f}\right) \end{array} \end{array}$$

Assume that the control design objective is to make the output of the plant follow a given, bounded, reference signal $q_{h,d}$, whose derivatives, $\overset{}{{\dot{q}}_{h,d,1}},\;{\ddot{q}}_{h,d,2},...,q_{h,d}^{\left(n\right)}$ are also bounded. Let, for the hip joint ${\left[q_{h,d,1},\;q_{h,d,2},\;q_{h,d,3}\right]}^{T}={\left[{\dot{q}}_{h,d},\;{\dot{q}}_{h,d,1},{\dot{q}}_{h,d,2}\right]}^{T}.$ Define $e_{i}=q_{h,d,i}-q_{i},\;i=1,2,...n$ and ${\tilde{q}}_{1}=q_{1}-{\hat{q}}_{1},\;{\tilde{q}}_{2}=q_{2}-\phi \left({\hat{q}}_{2},\alpha ,\delta \right)=q_{2}-{\hat{q}}_{2},\;{\tilde{q}}_{3}=q_{3}-{\hat{q}}_{3},e_{1}=q_{h,d,1}-q_{1},\;e_{2}=q_{h,d,2}-q_{2}$. The ADRC control law is given as

(29) $$\begin{array}{c} \begin{array}{c} \begin{array}{c} U_{1}=\left[k_{p}\left(q_{h,d,1}-{\hat{q}}_{1}\right)+k_{d}\left(q_{h,d,2}-{\hat{q}}_{2}\right)+q_{h,d,3}-{\hat{q}}_{3}\right]/D\left(1,1\right)\\ =\{[k_{p}[\left(q_{h,d,1}-\left(q_{1}-{\tilde{q}}_{1}\right)\right]+[k_{d}\left[\left(q_{h,d,2}-\left(q_{2}-{\tilde{q}}_{2}\right)\right]+q_{h,d,3}-{\hat{q}}_{3}\right\}/D\left(1,1\right)\\ =[k_{p}\left(e_{1}+{\tilde{q}}_{1}\right)+k_{d}\left[\left(e_{2}+{\tilde{q}}_{2}\right)+q_{d,3}-{\hat{q}}_{3}\right]/D\left(1,1\right) \end{array} \end{array} \end{array}$$

It follows that for the hip joint

(30) $$\begin{array}{c} \begin{array}{c} \begin{array}{c} \begin{array}{c} {\dot{e}}_{1}={\dot{q}}_{h,d,1}-{\dot{q}}_{1}=q_{h,d,2}-q_{2}=e_{2},\\ {\dot{e}}_{2}={\dot{q}}_{h,d,2}-{\dot{q}}_{2}=q_{h,d,3}-\left(q_{3}+D\left(1,1\right).U_{1}\right)\\ =q_{h,d,3}-q_{3}-\left[k_{p}\left(e_{1}+{\tilde{q}}_{1}\right)\right]-k_{d}\left[\left(e_{2}+{\tilde{q}}_{2}\right)-{\hat{q}}_{3}+q_{h,d,3}\right] \end{array}\\ =-k_{p}\left(e_{1}+{\tilde{q}}_{1}\right)-k_{d}[\left(e_{2}+{\tilde{q}}_{2}\right)-{\tilde{q}}_{3} \end{array} \end{array} \end{array}$$

Let $e={\left[e_{1},e_{2}\right]}^{T}\in R^{n},\;\tilde{q}={\left[{\tilde{q}}_{1},\;{\tilde{q}}_{2},\;{\tilde{q}}_{3}\right]}^{T}\in R^{n+1},$ then

(31) $$\begin{array}{c} \begin{array}{c} \begin{array}{c} \dot{e}\left(t\right)=A_{e}e\left(t\right)+A_{\tilde{q}}\;\tilde{q}\left(t\right)\\ A_{e}=\left[\begin{array}{cc} 0 & 1\\ -k_{p,h} & -k_{d,h} \end{array}\right]\;and\;A_{\tilde{q}}=\left[\begin{array}{ccc} 0 & 0 & 0\\ 0 & 0 & 0\\ -k_{p,h} & -k_{d,h} & -1 \end{array}\right] \end{array} \end{array} \end{array}$$

Similarly, for the knee joint

(32) $$\begin{array}{c} \begin{array}{c} \begin{array}{c} \dot{e}\left(t\right)=A_{e}e\left(t\right)+A_{\tilde{q}}\;\tilde{q}\left(t\right)\\ A_{e}=\left[\begin{array}{cc} 0 & 1\\ -k_{p,k} & -k_{d,k} \end{array}\right]\;and\;A_{\tilde{q}}=\left[\begin{array}{ccc} 0 & 0 & 0\\ 0 & 0 & 0\\ -k_{p,k} & -k_{d,k} & -1 \end{array}\right] \end{array} \end{array} \end{array}$$

Since $k_{p,h}$ and $k_{d,h}$ for the hip joint and $k_{p,h}$ and $k_{d,h}$ for the knee joint are selected in such a way that $s^{2}+k_{d}s+k_{p}$ is Hurwitz, $A_{e}$ is Hurwitz. For tuning simplicity we just let $s^{2}+k_{d}s+k_{p}={\left(s+\omega_{c}\right)}^{2}$ where $w_{c}>0$. This makes $w_{c}$, the controller bandwidth, only tuning parameter to be adjusted for controller.

$\lim_{t\to \infty }\left|\right|A_{\tilde{q}}\;\tilde{q}\left(t\right)\left|\right|=0$ if $h(q,u,w,\dot{w})$ is globally Lipschitz with respect to *q* [55]. For tuning simplicity $s^{2}+k_{d}s+k_{p}={\left(s+w_{c}\right)}^{2}$ where, $w_{c}>0$. This makes $w_{c}$ the only tuning parameter such that $\lim_{t\to \infty }e\left(t\right)=0,i=1,2,...,n$ Q.E.D. ${\tilde{q}}_{1},\;{\tilde{q}}_{2}\;{\tilde{q}}_{3},\;{\tilde{q}}_{4},\;{\tilde{q}}_{5}$, and ${\tilde{q}}_{6}$ are the observer estimation errors, $e_{1},\;e_{2},\;e_{3},\;e_{4},\;e_{5}$, and $e_{6}$ are the controller errors for hip joint and knee joints, respectively. From the above, it is shown that the closed loop system is asymptotically stable. □

## 6. Result Analysis and Discussion

The simulation studies for four cases are discussed. Case 1: without effect of disturbance, Case 2: with addition of random control disturbance, Case 3: with addition of constant control disturbance, and Case 4: with harmonic control disturbance. The parameters were chosen as shown in Table 4, for tracking differentiator design.

For NLSEF as shown in Table 5, the parameter values are selected. δ is the linear interval width of *fal*(·) [59] and relates to the error range. If it is too small, *fal*(·) will also cause the high-frequency flutter phenomenon.

Dr. Gao suggested factors to decide the bandwidth of the observer and controller [56], which has a vital effect on the performance of controller. A large bandwidth leads to noise sensitivity, a choice of which is a trade-off between the LESO performance and noise tolerance. For the designed LESO, the bandwidth of the observer is varied within the range of 400 rad/s to 1200 rad/s and the chosen value was 900 rad/s. The tracking error tends to decrease with the increase in observer bandwidth but it inversely affects the control effort which increases with the increase in bandwidth. The proportional and derivative gains in the ADRC was chosen by relation $k_{p}=w_{c}^{2}$ and $K_{d}=2w_{c}$ [56].

The proposed ADRC on LLRRE was tested by carrying out the simulation. All the simulations were performed with the sampling time 0.001 s and ode4 (Runge–Kutta) solver in MATLAB(*2019b*, *MathWorks*) [60]. In this paper, trajectories of the hip and knee joints are taken as predefined gait trajectory as a reference, external control disturbance of amplitude 5 N.m for constant disturbance and amplitude 5 N.m with frequency 50 Hz for a harmonic disturbance.

The performance indices chosen for comparison are Integral of the absolute magnitude of error (IAE), Integral time absolute error (ITAE), Integral square error (ISE), Integral time square error (ITSE), and Integral square of the control signal (ISU). All these performance indices can be formulated as:

(33) $$\begin{array}{c} \begin{array}{c} \begin{array}{c} IAE:\int_{0}^{t}\left|\left(r-y\right)\right|dt\\ ITAE:\int_{0}^{t}t\times \left|\left(r-y\right)\right|dt\\ \;ITSE:\int_{0}^{t}t\times {\left(r-y\right)}^{2}dt\\ \;ISE:\int_{0}^{t}{\left(r-y\right)}^{2}dt\\ \;ISU:\int_{0}^{t}{\left(u\right)}^{2}dt \end{array} \end{array} \end{array}$$

where, *r* is the reference input signal, *y* is output of the system, and r−y denotes the error of the system and *u* is the control output. IAE, ITAE, ISE, ITSE are known as time-integral criteria which are generic and comprehensive tools to evaluate the performance of a control system, they allow comparing between different controller designs or even different controller structure [61]. In this paper, the minimum value of index suggests best performance [62] and the parameters were chosen on that basis. Whereas ISU relates to denote control effort required for a controller [63]. The simulation results for gait trajectory tracking of LLRRE for the hip and knee joints, for various controllers, are compared. The comparison of conventional LESO based ADRC with proposed combinations ADRC-NLSEF, ADRC-TD, and ADRC-NLSEF-TD is carried out based performance parameters such as IAE, ITE, ITAE, ISTE, and ISU.

### 6.1. Effect of Disturbance

Case 1: No Disturbance

**Figure 4:**
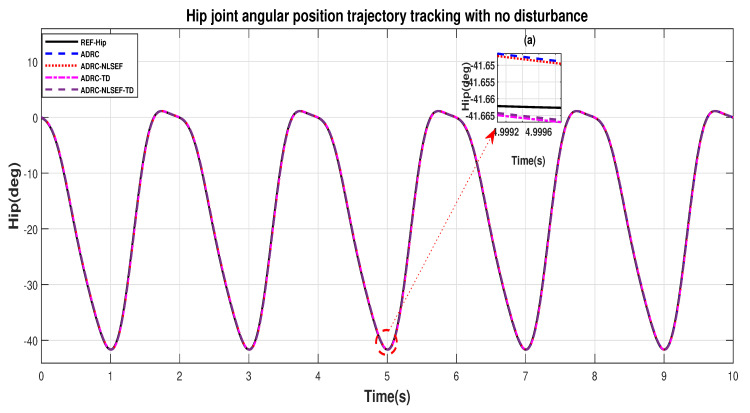
Gait trajectory tracking comparison of ADRC, ADRC-NLSEF, ADRC-TD, and ADRC-NLSEF-TD for the hip joint with a reference without disturbance.

**Figure 5:**
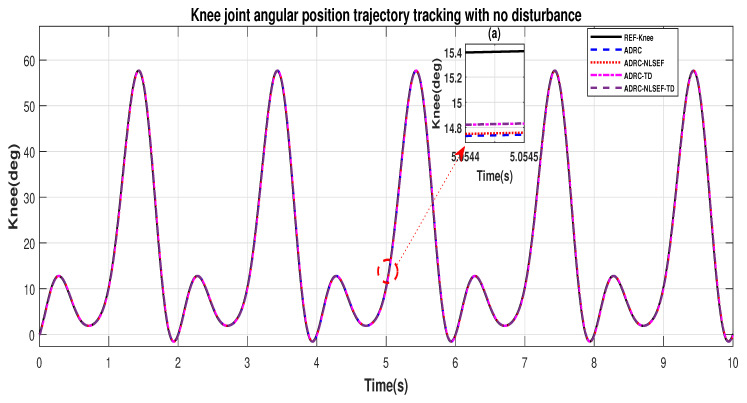
Gait trajectory tracking comparison of ADRC, ADRC-NLSEF, ADRC-TD, and ADRC-NLSEF-TD for the knee joint with a reference without disturbance.

**Figure 6:**
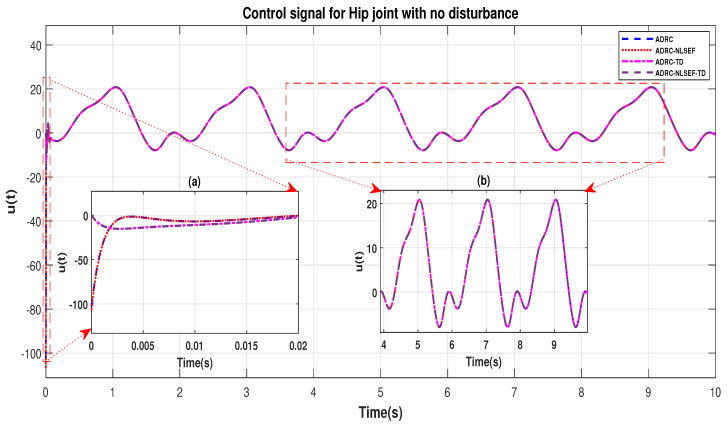
Control signal trajectory tracking comparison of ADRC, ADRC-NLSEF, ADRC-TD, and ADRC-NLSEF-TD for the hip joint with no disturbance. (**a**) initial response of the control signal; (**b**) control signal in blown up.

**Figure 7:**
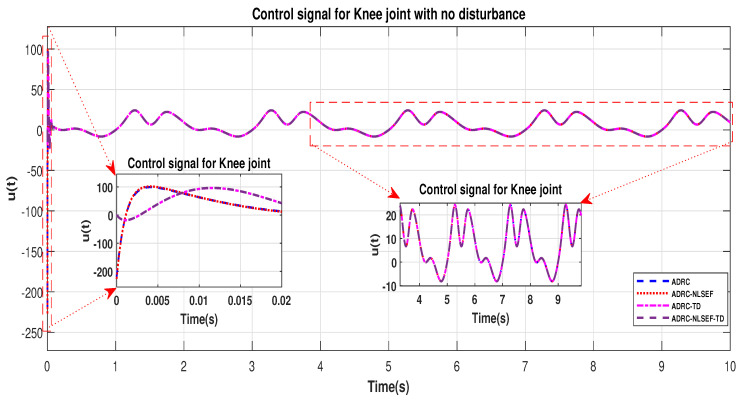
Control signal trajectory tracking comparison of ADRC, ADRC-NLSEF, ADRC-TD, and ADRC-NLSEF-TD for the knee joint with no disturbance.

**Figure 8:**
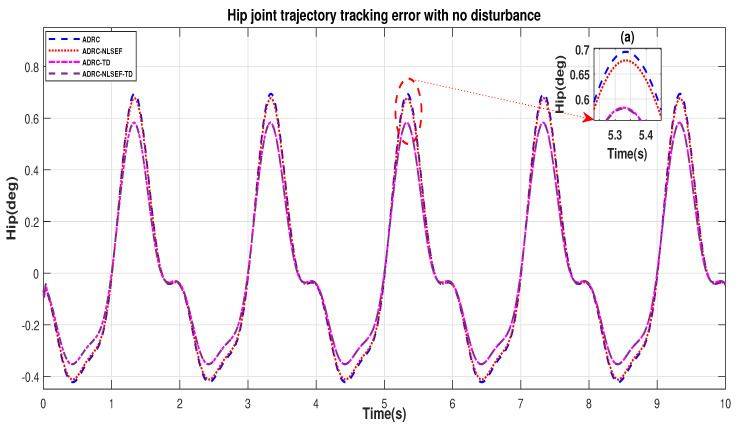
Gait trajectory tracking error comparison of ADRC, ADRC-NLSEF, ADRC-TD, and ADRC-NLSEF-TD for the hip joint with a reference without disturbance.

**Figure 9:**
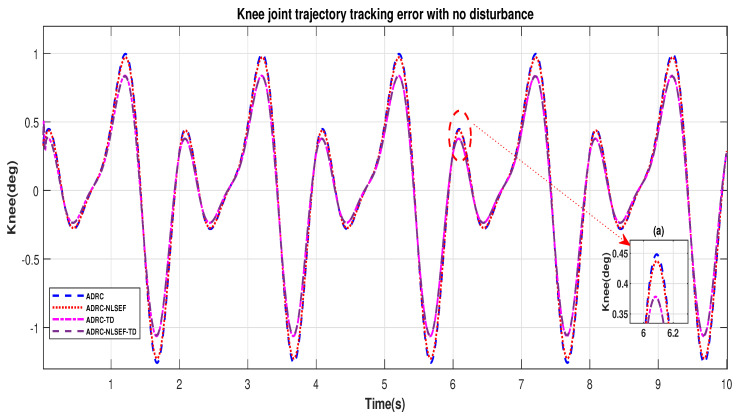
Gait trajectory tracking error comparison of ADRC, ADRC-NLSEF, ADRC-TD, and ADRC-NLSEF-TD for the knee joint with a reference without disturbance.

In Case 1, the performance of the ADRC is compared with the proposed controllers without external disturbance. Figure 4 and Figure 5 show the trajectory tracking performance of mentioned controllers for the hip and knee joints. Figure 6 and Figure 7 show the control signal required, and Figure 8 and Figure 9 show trajectory tracking error for ADRC, ADRC-NLSEF, ADRC-TD, and ADRC-NLSEF-TD for the hip and knee joints.

Figure 4 and Figure 5 show the trajectory tracking performance of the controllers in the no disturbance case. The trajectory tracked by ADRC-NLSEF-TD has best reference tracking followed by ADRC-TD, ADRC-NLSEF, and ADRC in the no disturbance case which can be seen from the minimized plot (a) in both the figures.

The initial response of the control signal is shown in the minimized plot (a) and control signal in blown up in the minimized plot (b) shown in Figure 6 and Figure 7, gives us the idea of control signal required by the controllers and no chattering in the control signal was observed in plot (b).

Figure 8 and Figure 9 show error while trajectory tracking in the minimized plot (a), in no disturbance case ADRC-NLSEF-TD outperforms all other controllers in terms of trajectory tracking followed by ADRC-TD, ADRC-NLSEF, and ADRC.

The Table 6 compares Performance indices for ADRC-NLSEF-TD, ADRC-TD, ADRC-NLSEF, and ADRC for the hip joint and the knee joint for no disturbance case.

ITSE, ISE, ITAE, IAE of ADRC-NLSEF-TD has values of 4.241, 0.8447, 11.85, and 2.397 for the hip joints and 13.2, 2.454, 20.3, and 3.883 for the knee joints, respectively which are minimum as compared ADRC-TD, ADRC-NLSEF, ADRC. This proves that In terms of trajectory tracking ADRC-NLSEF-TD has better performance. ADRC has ISU 1239 for the hip joint and 1722 for the knee joint which is almost the same or a slightly less than ADRC-TD, ADRC-NLSEF, and ADRC-NLSEF-TD.

Case 2: Random Disturbance

**Figure 10:**
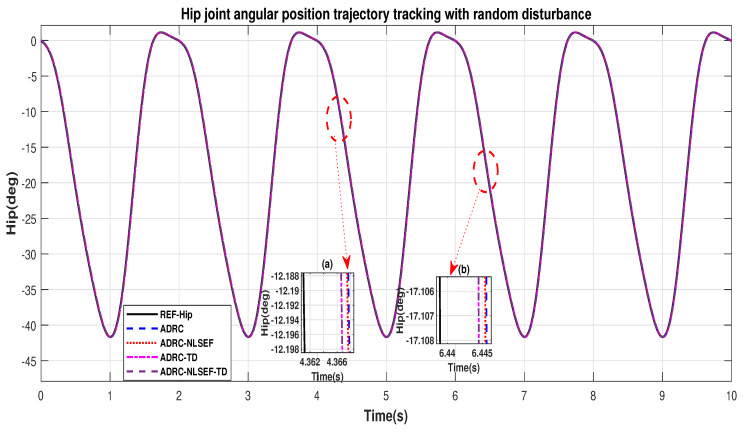
Gait trajectory tracking comparison of ADRC, ADRC-NLSEF, ADRC-TD, and ADRC-NLSEF-TD for the hip joint with a reference with random disturbance.

**Figure 11:**
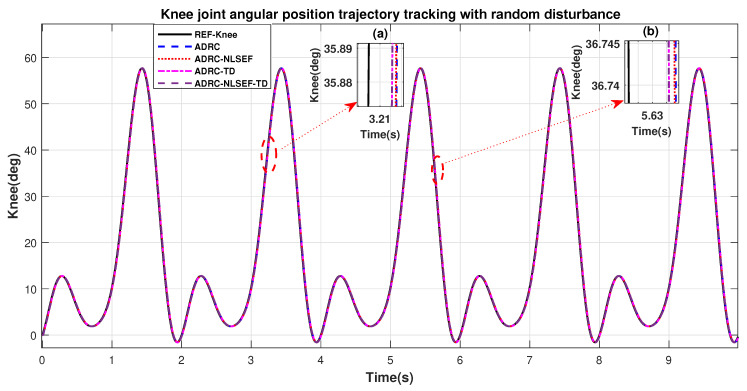
Gait trajectory tracking comparison of ADRC, ADRC-NLSEF, ADRC-TD, and ADRC-NLSEF-TD for the knee joint with a reference with random disturbance.

**Figure 12:**
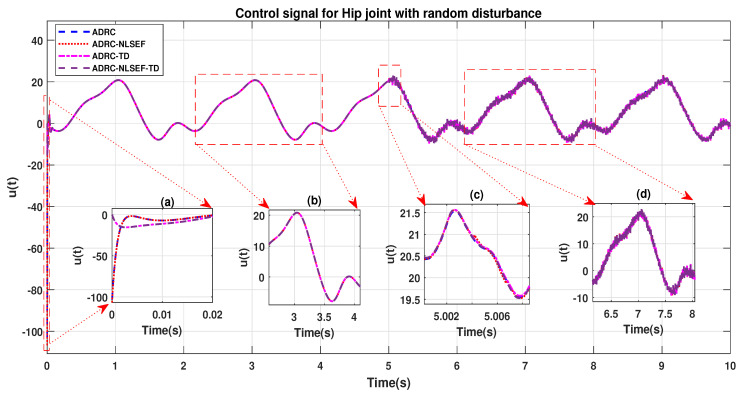
Control signal trajectory tracking comparison of ADRC, ADRC-NLSEF, ADRC-TD, and ADRC-NLSEF-TD for the hip joint with random disturbance.

**Figure 13:**
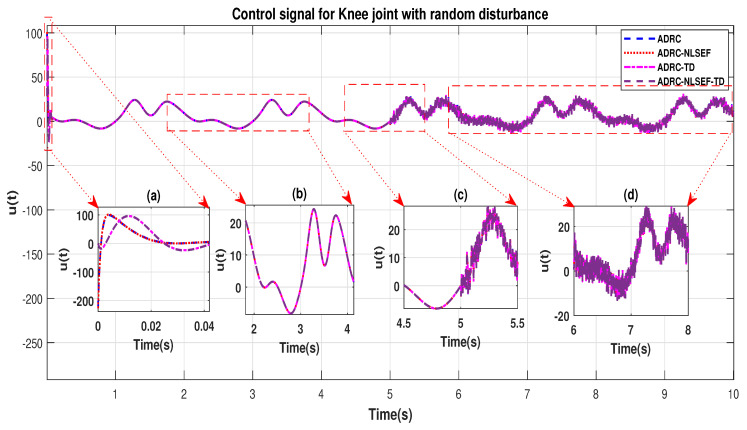
Control signal trajectory tracking comparison of ADRC, ADRC-NLSEF, ADRC-TD, and ADRC-NLSEF-TD for the knee joint with random disturbance.

**Figure 14:**
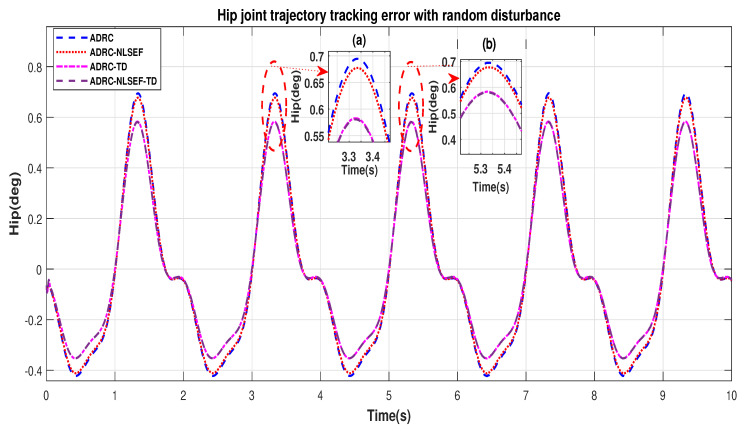
Gait trajectory tracking error comparison of ADRC, ADRC-NLSEF, ADRC-TD, and ADRC-NLSEF-TD for the hip joint for reference signal with random disturbance.

**Figure 15:**
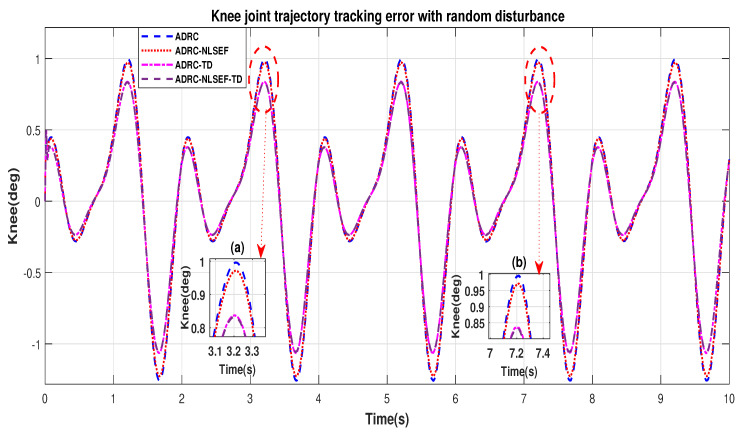
Gait trajectory tracking error comparison of ADRC, ADRC-NLSEF, ADRC-TD, and ADRC-NLSEF-TD for the knee joint for reference signal with random disturbance.

In Case 2, The performance of the ADRC is compared with the proposed controllers with addition of random control disturbance (between −1 and 1) *N.m*. The sampling time is 0.001 s. *N.m*. at *t* = 5 s. Figure 10 and Figure 11 show the trajectory tracking performance of mentioned controllers for the hip and knee joints. Figure 12 and Figure 13 show the control signal required and Figure 14 and Figure 15 show the trajectory tracking error or ADRC, ADRC-NLSEF, ADRC-TD, and ADRC-NLSEF-TD for the hip and knee joints.

Figure 10 and Figure 11 show the trajectory tracking performance for various controllers. The minimized plot (a), shows the trajectory tracking response of the controllers before the introduction of the random disturbance. Plot (b) shows the controller trajectory tracking performance after addition of random disturbance at *t* = 5 s. The trajectory tracked by ADRC-NLSEF-TD has best reference tracking followed by ADRC-TD, ADRC-NLSEF, and ADRC before and after inclusion of disturbance at *t* = 5 s, which proves its effectiveness against random disturbance.

The initial response of the control signal is shown in the minimized plot (a) and control signal before inclusion of random disturbance in blown up in the minimized plot (b) shown in Figure 12 and Figure 13. Plot (c) and plot (d) show the effect of random disturbance at *t* = 5 s and after 5 s.

Figure 14 and Figure 15 show the error trajectory generated while reference tracking, the performance of the controller before and after addition of the random disturbance can be seen from the minimized plot (a) and plot (b), concludes the superiority of ADRC-NLSEF-TD over other controllers of trajectory tracking before and after addition of random disturbance.

Table 7 compares performance indices for ADRC-NLSEF-TD, ADRC-TD, ADRC-NLSEF, and ADRC for the hip joint and the knee joint for random disturbance case.

ITSE, ISE, ITAE, IAE of ADRC-NLSEF-TD values are 4.241, 0.8447, 11.85, and 2.397 for the hip joints and 13.2, 2.454, 20.3, and 3.883 for the knee joints, respectively which are minimum as compared to ADRC-TD, ADRC-NLSEF, ADRC. This proves that In terms of trajectory tracking ADRC-NLSEF-TD has better performance compared to the rest of the controllers. ADRC has ISU 1299 for the hip joint and 1801 for the knee joint which is almost the same or a slightly less than ADRC-TD, ADRC-NLSEF, and ADRC-NLSEF-TD.

Case 3: Constant Disturbance

**Figure 16:**
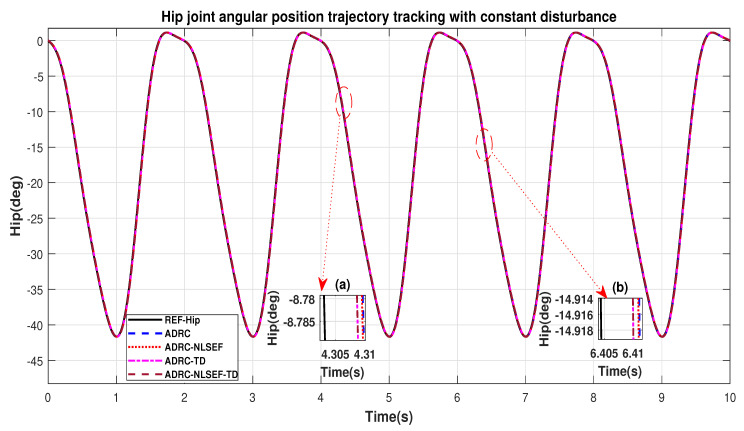
Gait trajectory tracking comparison of ADRC, ADRC-NLSEF, ADRC-TD, and ADRC-NLSEF-TD for the hip joint with a reference with constant disturbance.

**Figure 17:**
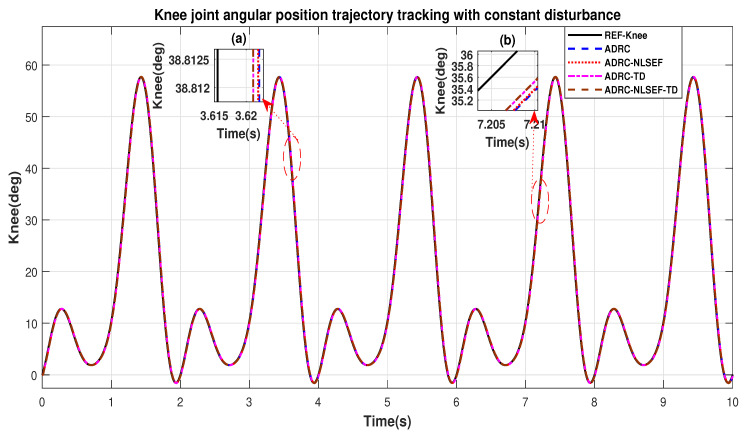
Gait trajectory tracking comparison of ADRC, ADRC-NLSEF, ADRC-TD, and ADRC-NLSEF-TD for the knee joint with a reference with constant disturbance.

**Figure 18:**
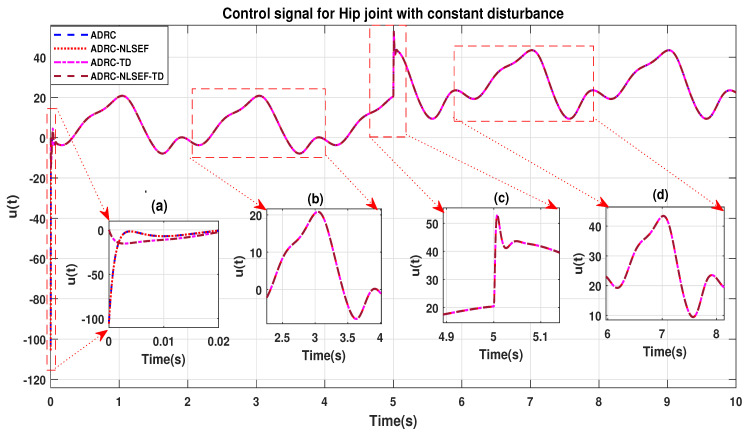
Control signal comparison of ADRC, ADRC-NLSEF, ADRC-TD, and ADRC-NLSEF-TD for the hip joint with constant disturbance.

**Figure 19:**
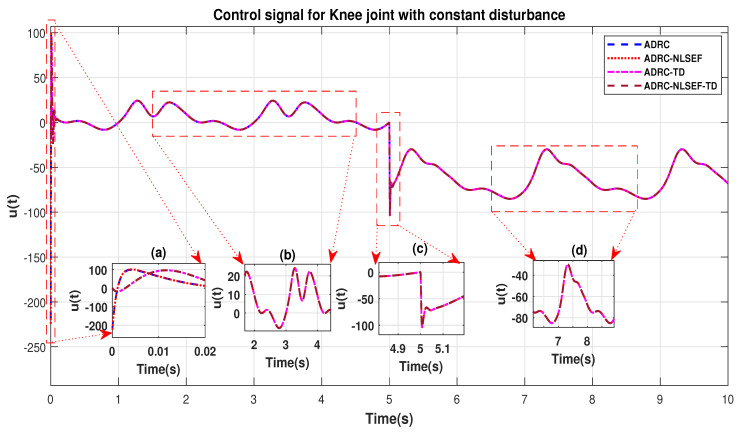
Control signal comparison of ADRC, ADRC-NLSEF, ADRC-TD, and ADRC-NLSEF-TD for knee Joint with constant disturbance.

**Figure 20:**
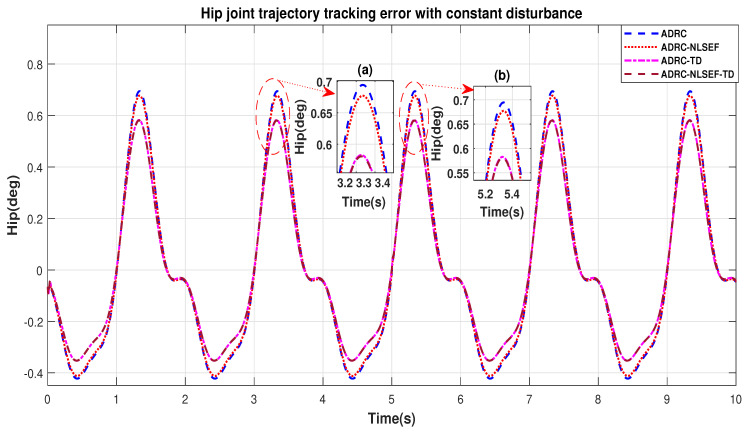
Gait trajectory tracking error comparison of ADRC, ADRC-NLSEF, ADRC-TD, and ADRC-NLSEF-TD for the hip joint with a reference with constant disturbance.

**Figure 21:**
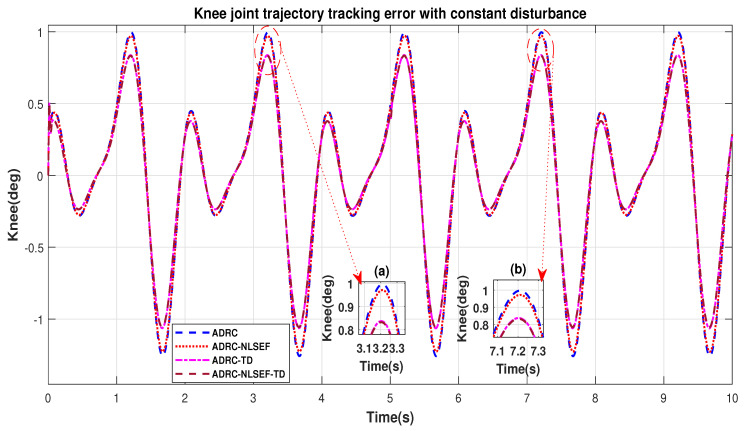
Gait trajectory tracking error comparison of ADRC, ADRC-NLSEF, ADRC-TD, and ADRC-NLSEF-TD for knee joint with a reference with constant disturbance.

In Case 3, the performance of the ADRC is compared with the proposed controllers with addition of constant control disturbance of amplitude 5 N.m. at *t* = 5 s. Figure 16 and Figure 17 shows the trajectory tracking performance for various controllers for the hip and knee joints. Figure 18 and Figure 19 show the control signal required. Figure 20 and Figure 21 show tracking error for ADRC, ADRC-NLSEF, ADRC-TD, and ADRC-NLSEF-TD for the hip and knee joints with constant disturbance.

Figure 16 and Figure 17 show the trajectory tracking performance for various controllers. The minimized plot (a), show the trajectory tracking response of the controllers before the introduction of the constant disturbance. Plot (b) shows the controller trajectory tracking performance after addition of constant disturbance at *t* = 5 s. The trajectory tracked by ADRC-NLSEF-TD has best reference tracking followed by ADRC-TD, ADRC-NLSEF, and ADRC before and after inclusion of disturbance at *t* = 5 s, which proves its effectiveness against constant disturbance.

The initial response of the control signal is shown in the minimized plot (a) and control signal before inclusion of random disturbance in blown up in the minimized plot (b) shown in Figure 18 and Figure 19. Plot (c) and plot (d) show the effect of constant disturbance at *t* = 5 s and after 5 s.

Figure 20 and Figure 21 show the error trajectory generated while reference tracking, the performance of the controller before and after addition of the constant disturbance can be seen from the minimized plot (a) and plot (b), concluding the superiority of ADRC-NLSEF-TD over other controllers of trajectory tracking before and after addition of constant disturbance.

Table 8 compares performance indices for ADRC-NLSEF-TD, ADRC-TD, ADRC-NLSEF, and ADRC for the hip joint and the knee joint for the constant disturbance case.

ITSE, ISE, ITAE, IAE of ADRC-NLSEF-TD had values of 4.241, 0.8446, 11.85, and 2.397 for hip joints and 13.21, 2.456, 20.31, and 3.884 for knee joints, respectively which are minimum as compared to ADRC-TD, ADRC-NLSEF, ADRC. This proves that in terms of trajectory tracking, ADRC-NLSEF-TD has better performance compared to the rest of the controllers. ADRC has ISU 6214 for the hip joint and 3.166 ×$10^{4}$ for the knee joint which is almost the same or a slightly less than ADRC-TD, ADRC-NLSEF, and ADRC-NLSEF-TD.

Case 4: Harmonic Disturbance

**Figure 22:**
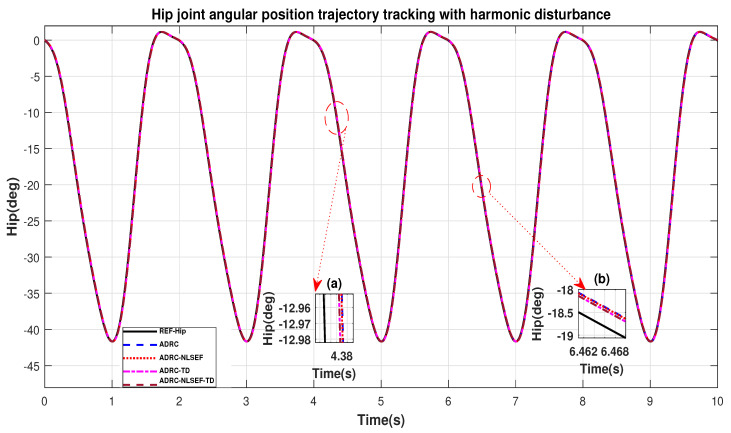
Gait trajectory tracking comparison of ADRC, ADRC-NLSEF, ADRC-TD, and ADRC-NLSEF-TD for the hip joint with a reference with harmonic disturbance.

**Figure 23:**
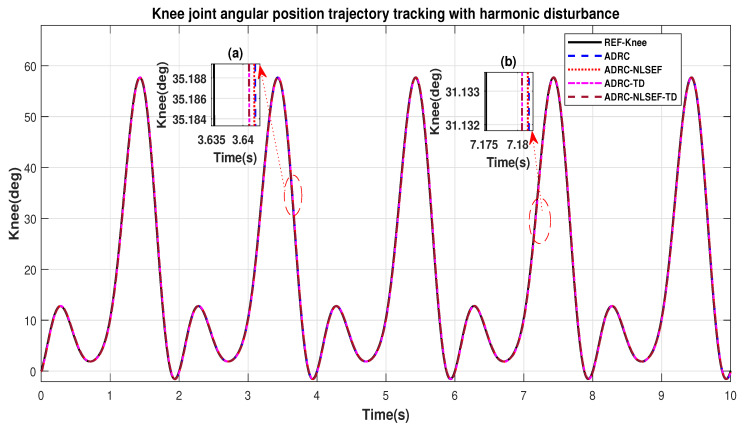
Gait trajectory tracking comparison of ADRC, ADRC-NLSEF, ADRC-TD, and ADRC-NLSEF-TD for the knee joint with a reference with harmonic disturbance.

**Figure 24:**
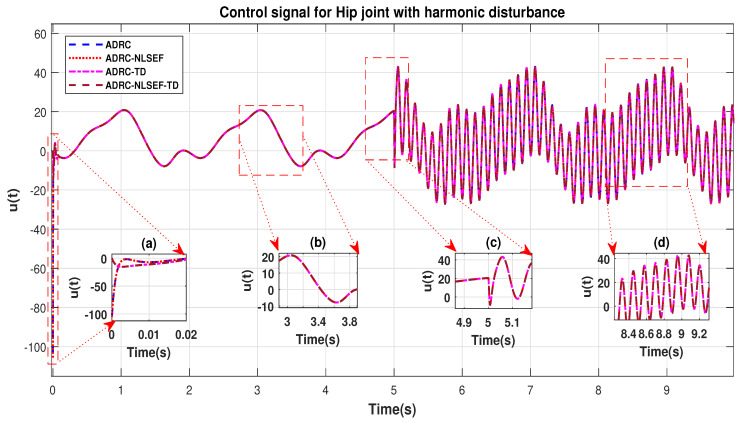
Control signal comparison of ADRC, ADRC-NLSEF, ADRC-TD, and ADRC-NLSEF-TD for the hip joint with harmonic disturbance.

**Figure 25:**
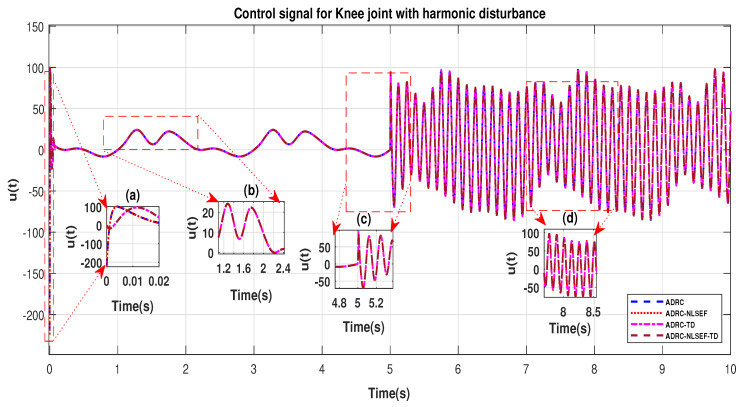
Control signal comparison of ADRC, ADRC-NLSEF, ADRC-TD, and ADRC-NLSEF-TD for the knee joint with harmonic disturbance.

**Figure 26:**
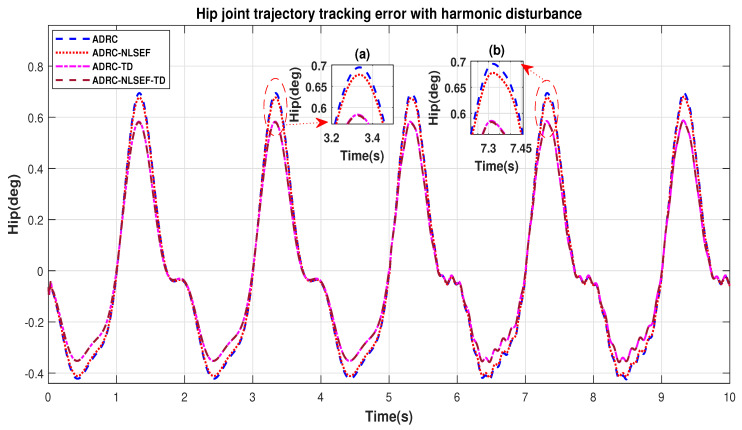
Gait trajectory tracking error comparison of ADRC, ADRC-NLSEF, ADRC-TD, and ADRC-NLSEF-TD for the hip joint with a reference with harmonic disturbance.

**Figure 27:**
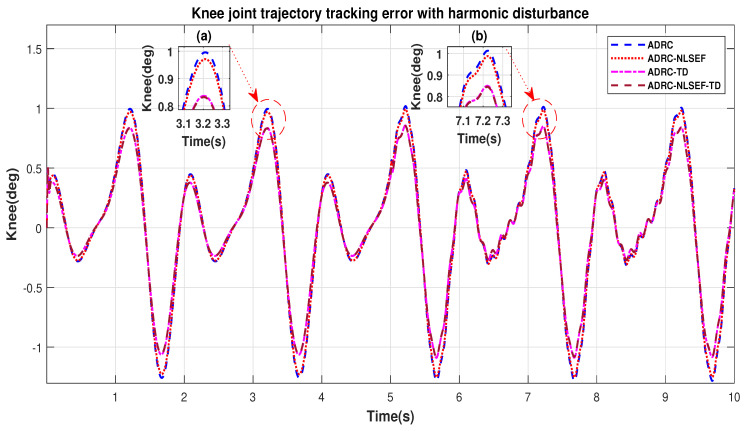
Gait trajectory tracking error comparison of ADRC, ADRC-NLSEF, ADRC-TD, and ADRC-NLSEF-TD for the knee joint with a reference with harmonic disturbance.

In Case 4, the performance of the ADRC is compared with the proposed controllers with addition of harmonic control disturbance of amplitude 5 *N.m. at t* = 5 s. Figure 22 and Figure 23 shows the trajectory tracking performance for various controllers. Figure 24 and Figure 25 show control signal required. Figure 26 and Figure 27 show tracking error for ADRC, ADRC-NLSEF, ADRC-TD, and ADRC-NLSEF-TD for the hip and knee joints with harmonic disturbance.

Figure 22 and Figure 23 show the trajectory tracking performance for various controllers. The minimized plot (a), show the trajectory tracking response of the controllers before the introduction of the harmonic disturbance. Plot (b) show the controller trajectory tracking performance after addition of harmonic disturbance at *t* = 5 s. The trajectory tracked by ADRC-NLSEF-TD has the best reference tracking followed by ADRC-TD, ADRC-NLSEF, and ADRC before and after inclusion of disturbance at *t* = 5 s, which proves its effectiveness against harmonic disturbance.

The initial response of the control signal is shown in the minimized plot (a) and control signal before inclusion of harmonic disturbance in blown up in the minimized plot (b) shown in Figure 24 and Figure 25. Plot (c) and plot (d) show the effect of harmonic disturbance at *t* = 5 s and after 5 s.

Figure 26 and Figure 27 show the error trajectory generated while reference tracking, the performance of the controller before and after addition of the harmonic disturbance can be seen from the minimized plot (a) and plot (b), concludes the superiority of ADRC-NLSEF-TD over other controllers of trajectory tracking before and after addition of harmonic disturbance.

The Table 9 compares Performance indices for ADRC-NLSEF-TD, ADRC-TD, ADRC-NLSEF, and ADRC for the hip joint and the knee joint for no disturbance case.

ITSE, ISE, ITAE, IAE of ADRC-NLSEF-TD had values of 4.243, 0.8450, 11.86, and 2.399 for the hip joints and 13.22, 2.457, 20.35, and 3.89 for the knee joints, respectively which are minimum as compared to ADRC-TD, ADRC-NLSEF, ADRC. This proves that In terms of trajectory tracking ADRC-NLSEF-TD has better performance compared to all other controllers. ADRC has ISU 3037 for the hip joint and 2.039$\times 10^{4}$ for the knee joint which is almost the same or a slightly less than ADRC-TD, ADRC-NLSEF, and ADRC-NLSEF-TD.

Overall Comparison of Four Disturbance Cases

Table 10 shows the overall performance of four cases for various combinations such as ADRC, ADRC-NLSEF, ADRC-TD, and ADRC-NLSEF-TD for the hip joint.

Table 11 shows the overall performance of four cases for various combinations such as ADRC, ADRC-NLSEF, ADRC-TD, and ADRC-NLSEF-TD for the knee joint.

It was observed that there is almost no change in any of the performance indices other than a slight change in ISU, which indicates the increase in control effort. This change is significant in case of constant disturbance when compared to other disturbance cases. Amongst all the controllers ADRC-NLSEF-TD proves a better selection because of its best tracking capabilities followed by ADRC-TD and ADRC-NLSEF, prove it as a promising strategy. The proposed controller ADRC-NLSEF-TD was found to provide a better performance in comparison to only LESO based ADRC i.e., ADRC.

### 6.2. Effect of Parameter Variation

To demonstrate the efficiency of proposed strategy over conventional LESO based ADRC, ±20% parameter variations are included in this subsection. The parameters are varied from actual values are given in Table 12, *g* is kept constant as 9.81 m/s${}^{2}$ following observation are obtained are listed in Table 13, Table 14, Table 15 and Table 16.

The model parameters are varied with ±20% variations. The results are obtained for the gait trajectory tracking for the hip and knee joints, based on the performance indices. It can be concluded from the above results that the proposed control method performs superior and tracks the trajectory efficiently as compared to ADRC even with parameter variation the only change in the ISU occurs which defines the control effort, it is observed that the control effort with decreases with +20% variation and increases with −20% parametric variation.

### 6.3. Effect of Noise

The sinusoidal noise of −0.5° to 0.5° variance is incorporated with the +20% parameter variation and under the influence of various disturbance to demonstrate the efficacy of proposed controller with actuator saturation. Generally, the larger the observer bandwidth is, the more accurate the estimation will be. However, a large observer bandwidth will increase noise sensitivity. Therefore, a proper observer bandwidth should be selected in a compromise between the tracking performance and noise tolerance. The performance under noise is tested with four disturbance cases and analyzed based on the performance indices.

Case 1: No Disturbance

**Figure 28:**
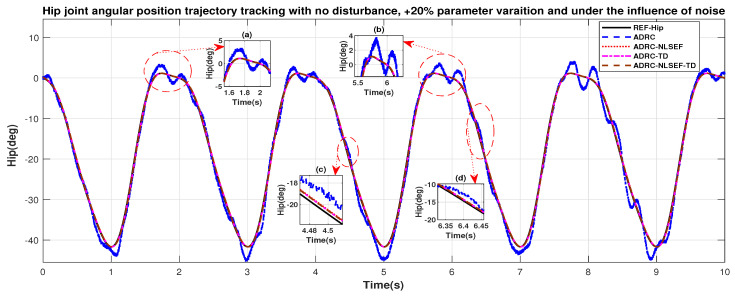
Gait trajectory tracking comparison of ADRC, ADRC-NLSEF, ADRC-TD, and ADRC-NLSEF-TD for the hip joint under the influence of noise, with parameter variation and without disturbance effect.

**Figure 29:**
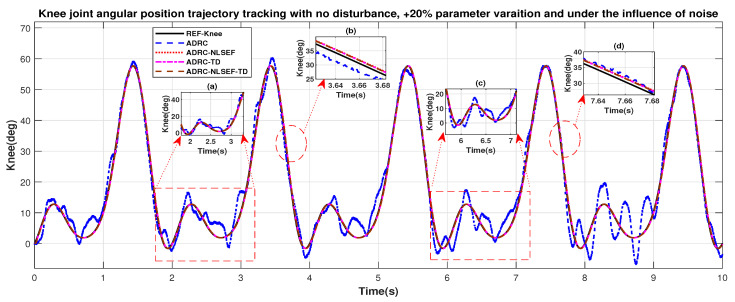
Gait trajectory tracking comparison of ADRC, ADRC-NLSEF, ADRC-TD, and ADRC-NLSEF-TD for the knee joint under the influence of noise, with parameter variation and without disturbance effect.

**Figure 30:**
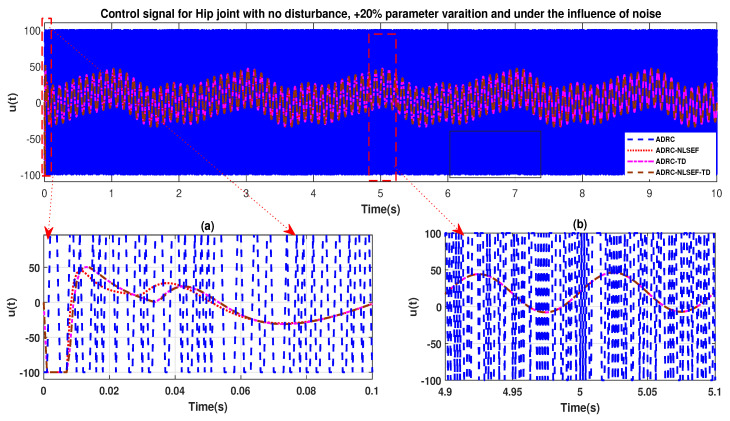
Control signal trajectory tracking comparison of ADRC, ADRC-NLSEF, ADRC-TD, and ADRC-NLSEF-TD for the hip joint under the influence of noise, with parameter variation and without disturbance effect.

**Figure 31:**
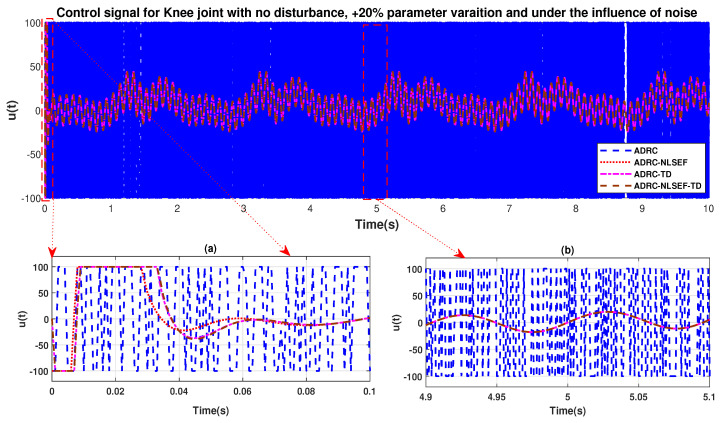
Control signal trajectory tracking comparison of ADRC, ADRC-NLSEF, ADRC-TD, and ADRC-NLSEF-TD for the knee joint under the influence of noise, with parameter variation and without disturbance effect.

**Figure 32:**
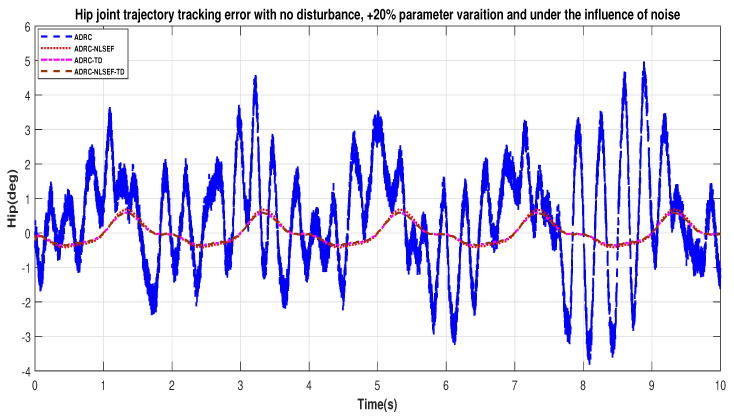
Gait trajectory tracking error comparison of ADRC, ADRC-NLSEF, ADRC-TD, and ADRC-NLSEF-TD for the hip joint under the influence of noise, with parameter variation and without disturbance effect.

**Figure 33:**
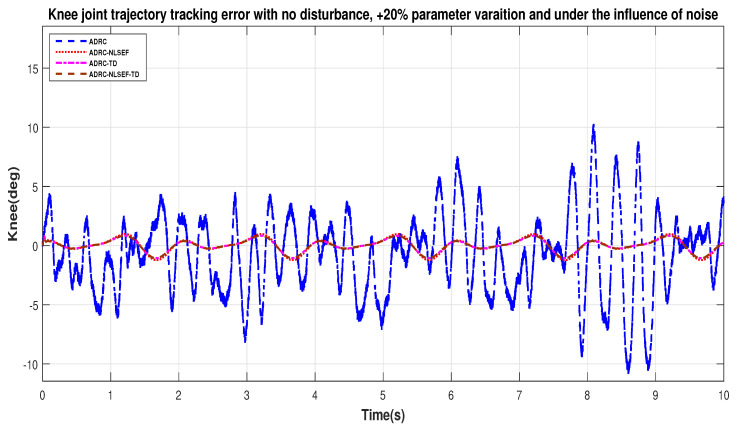
Gait trajectory tracking error comparison of ADRC, ADRC-NLSEF, ADRC-TD, and ADRC-NLSEF-TD for the knee joint under the influence of noise, with parameter variation and without disturbance effect.

In Case 1, the performance of the ADRC is compared with the proposed controllers with the only effect of noise and +20% parametric variation without the addition of external control disturbance. Figure 28 and Figure 29 shows the trajectory tracking performance of mentioned controllers for the hip and knee joints. Figure 30 and Figure 31 show control signal required, and Figure 32 and Figure 33 show trajectory tracking error for ADRC, ADRC-NLSEF, ADRC-TD, and ADRC-NLSEF-TD for the hip and knee joints. Figure 28 and Figure 29 show the trajectory tracking performance of the controllers in no disturbance case. The trajectory tracked by ADRC-NLSEF-TD has best reference tracking followed by ADRC-TD, ADRC-NLSEF, and ADRC in no disturbance case which can be seen from the minimized plot (a), (b), (c), and (d) in both the figures.

The initial response of the control signal is shown in the minimized plot (a) and control signal in blown up in the minimized plot (b) shown in Figure 30 and Figure 31, gives us the idea of control signal required by the controllers. The control signal for ADRC completely saturates whereas in the proposed controlled methods it firstly saturates for a while and maintains its safe limit.

Figure 32 and Figure 33 show error while trajectory tracking under the influence of noise, with parameter variation and without disturbance effect. ADRC-NLSEF-TD outperforms all other controllers in terms of trajectory tracking followed by ADRC-TD, ADRC-NLSEF, and ADRC.

Case 2: Random Disturbance

**Figure 34:**
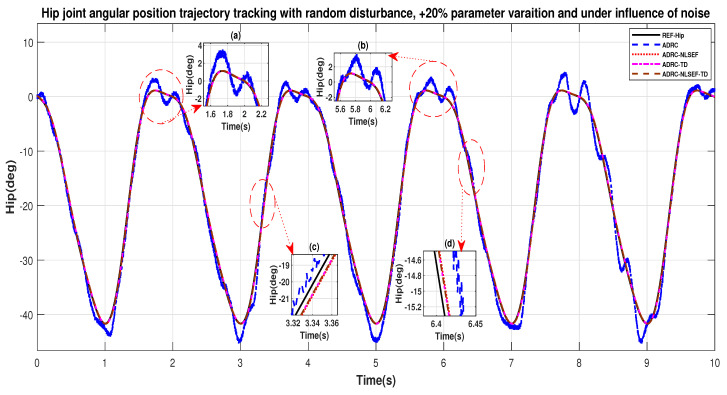
Gait trajectory tracking comparison of ADRC, ADRC-NLSEF, ADRC-TD, and ADRC-NLSEF-TD for the hip joint under the influence of noise, with parameter variation and with random disturbance effect.

**Figure 35:**
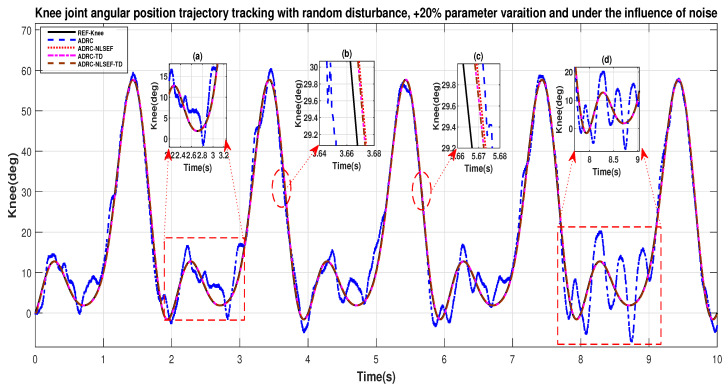
Gait trajectory tracking comparison of ADRC, ADRC-NLSEF, ADRC-TD, and ADRC-NLSEF-TD for the knee joint under the influence of noise, with parameter variation and with random disturbance effect.

**Figure 36:**
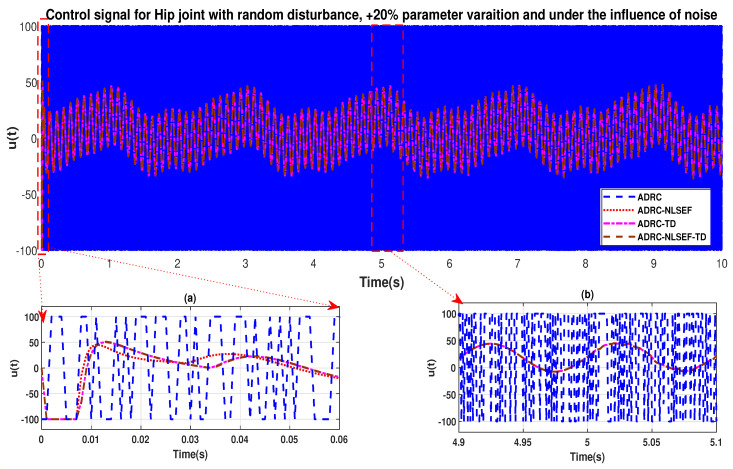
Control signal trajectory tracking comparison of ADRC, ADRC-NLSEF, ADRC-TD, and ADRC-NLSEF-TD for the knee joint under the influence of noise, with parameter variation and with random disturbance effect.

**Figure 37:**
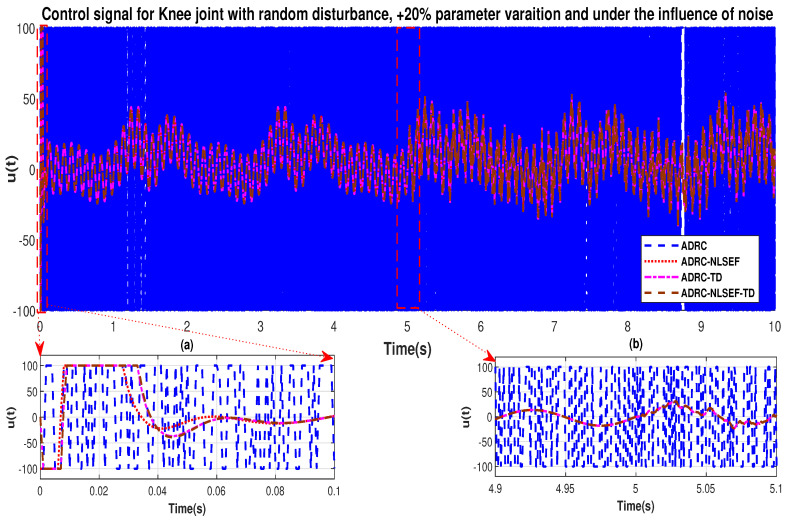
Control signal trajectory tracking comparison of ADRC, ADRC-NLSEF, ADRC-TD, and ADRC-NLSEF-TD for the knee joint under the influence of noise, with parameter variation and with random disturbance effect.

**Figure 38:**
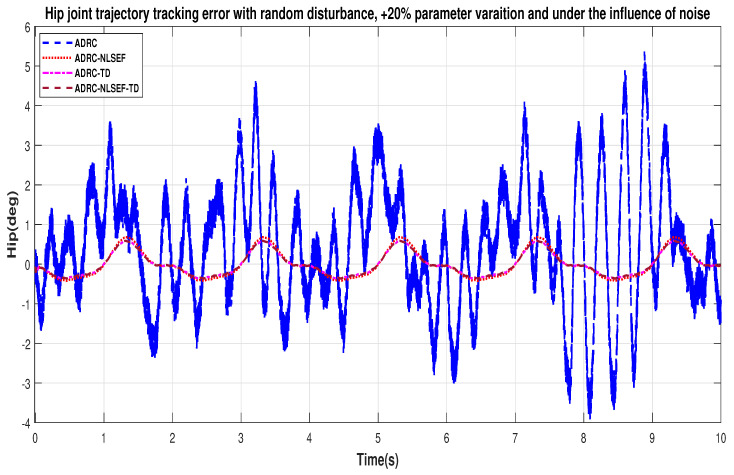
Gait trajectory tracking error comparison of ADRC, ADRC-NLSEF, ADRC-TD, and ADRC-NLSEF-TD for the hip joint under the influence of noise, with parameter variation and with random disturbance effect.

**Figure 39:**
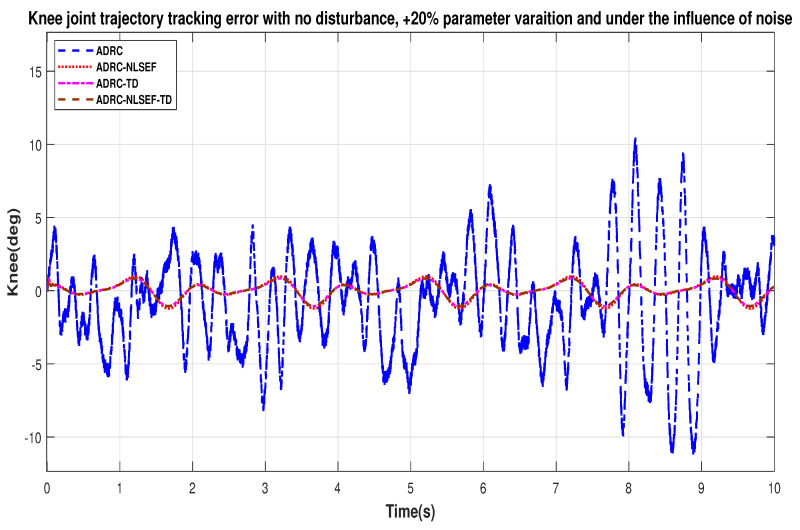
Gait trajectory tracking error comparison of ADRC, ADRC-NLSEF, ADRC-TD, and ADRC-NLSEF-TD for the hip joint under the influence of noise, with parameter variation and with random disturbance effect.

In Case 2, the performance of the ADRC is compared with the proposed controllers under the influence of noise, parametric variation of and with the addition of random control disturbance (between −1 and 1) *N.m*. The sampling time is 0.001 s. *N.m*. at *t* = 5 s. Figure 34 and Figure 35 show the trajectory tracking performance of mentioned controllers for the hip and knee joints. Figure 36 and Figure 37 show the control signal required and, Figure 38 and Figure 39 show the trajectory tracking error or ADRC, ADRC-NLSEF, ADRC-TD, and ADRC-NLSEF-TD for the hip and knee joints.

Figure 34 and Figure 35 show the trajectory tracking performance for various controllers. The minimized plot (a) and plot (b) show the trajectory tracking response of the controllers before the introduction of the random disturbance. Plot (c) and plot (d) show the controller trajectory tracking performance after addition of random disturbance at *t* = 5 s. The trajectory tracked by ADRC-NLSEF-TD has the best reference tracking followed by ADRC-TD, ADRC-NLSEF, and ADRC before and after inclusion of disturbance at *t* = 5 s, which prove its effectiveness under the influence of noise, parametric variation, and against random disturbance.

The initial response of the control signal is shown in the minimized plot (a) and control signal in blown up in the minimized plot (b) shown in Figure 36 and Figure 37, gives us the idea of control signal required by the controllers. The control signal for ADRC completely saturates whereas in the proposed controlled methods it firstly saturates for a while and maintains its safe limit. Plot (b) show the effect of random disturbance at *t* = 5 s and after 5 s.

Figure 38 and Figure 39 show the error trajectory generated while reference tracking, the performance of the controller before and after addition of the random disturbance, concludes the superiority of ADRC-NLSEF-TD over other controllers of trajectory tracking before and after addition of random disturbance, under the influence of noise and with parameter variation.

Case 3: Constant Disturbance

**Figure 40:**
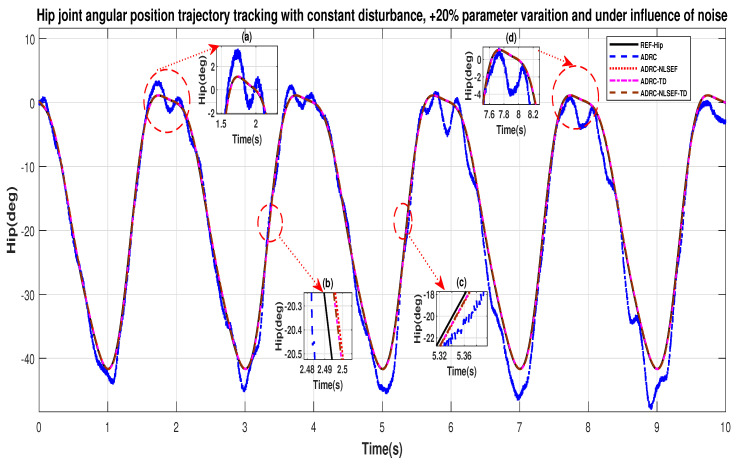
Gait trajectory tracking comparison of ADRC, ADRC-NLSEF, ADRC-TD, and ADRC-NLSEF-TD for knee joint under the influence of noise, with parameter variation and with constant disturbance effect.

**Figure 41:**
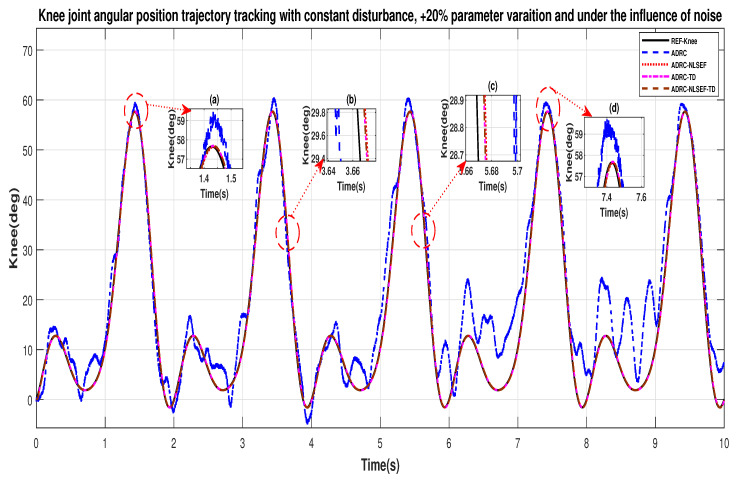
Gait trajectory tracking comparison of ADRC, ADRC-NLSEF, ADRC-TD, and ADRC-NLSEF-TD for the knee joint under the influence of noise, with parameter variation and with constant disturbance effect.

**Figure 42:**
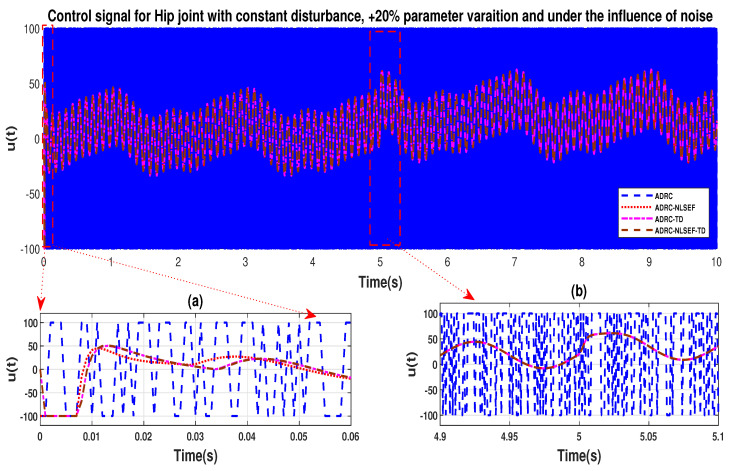
Control signal trajectory tracking comparison of ADRC, ADRC-NLSEF, ADRC-TD, and ADRC-NLSEF-TD for the knee joint under the influence of noise, with parameter variation and with constant disturbance effect.

**Figure 43:**
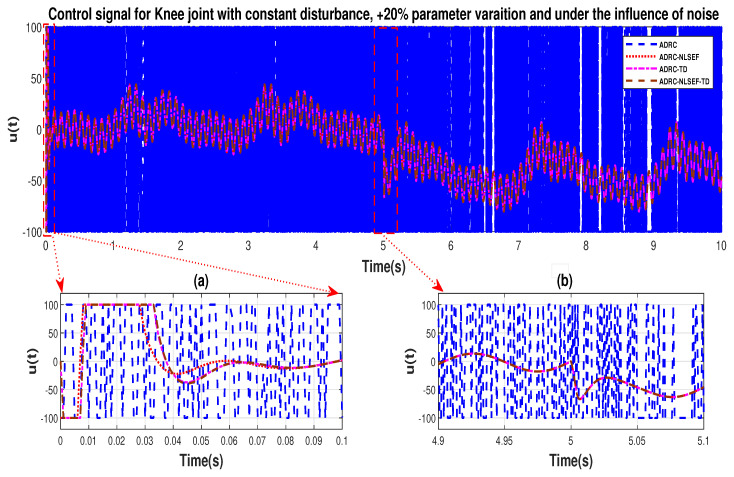
Control signal trajectory tracking comparison of ADRC, ADRC-NLSEF, ADRC-TD, and ADRC-NLSEF-TD for the knee joint under the influence of noise, with parameter variation and with constant disturbance effect.

**Figure 44:**
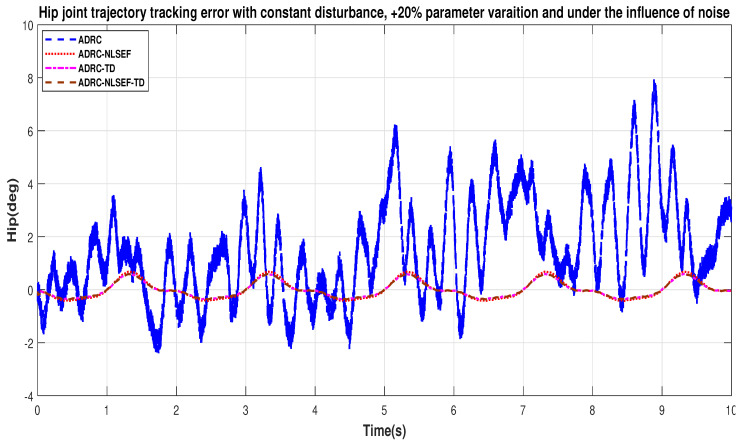
Gait trajectory tracking error comparison of ADRC, ADRC-NLSEF, ADRC-TD, and ADRC-NLSEF-TD for hip joint under the influence of noise, with parameter variation and with constant disturbance effect.

**Figure 45:**
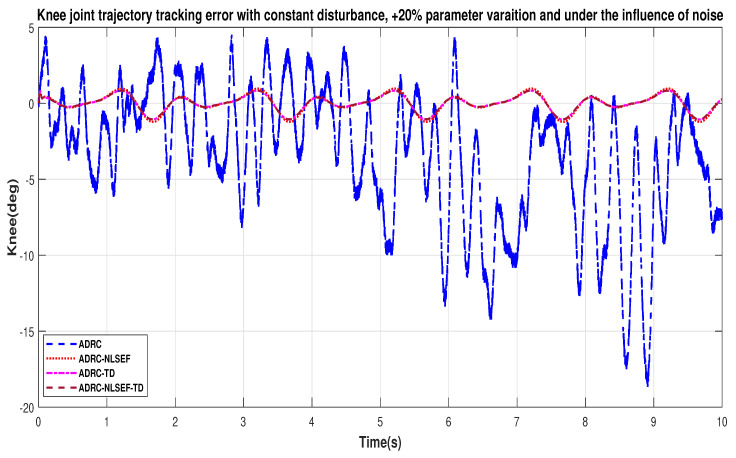
Gait trajectory tracking error comparison of ADRC, ADRC-NLSEF, ADRC-TD, and ADRC-NLSEF-TD for hip joint under the influence of noise, with parameter variation and with constant disturbance effect.

In Case 3, the performance of the ADRC is compared with the proposed controllers under the influence of noise, with parametric variation and with addition of constant control disturbance of amplitude *5 N.m.* at *t* = 5 s. Figure 40 and Figure 41 show the trajectory tracking performance for various controllers for the hip and knee joints. Figure 42 and Figure 43 show the control signal required. Figure 44 and Figure 45 show tracking error for ADRC, ADRC-NLSEF, ADRC-TD, and ADRC-NLSEF-TD for the hip and knee joints with constant disturbance.

Figure 40 and Figure 41 show the trajectory tracking performance for various controllers. The minimized plot (a) and plot (b), show the trajectory tracking response of the controllers before the introduction of the constant disturbance. Plot (c) and plot (d) show the controller trajectory tracking performance after addition of constant disturbance at *t* = 5 s. The trajectory tracked by ADRC-NLSEF-TD has best reference tracking followed by ADRC-TD, ADRC-NLSEF, and ADRC before and after inclusion of disturbance at *t* = 5 s, which proves its effectiveness under the influence of noise, parametric variation and against constant disturbance.

The initial response of the control signal is shown in the minimized plot (a) and control signal in blown up in the minimized plot (b) shown in Figure 42 and Figure 43, gives us the idea of control signal required by the controllers. The control signal for ADRC completely saturates whereas in the proposed controlled methods it firstly saturates for a while and maintains its safe limit. Plot (b) show the effect of constant disturbance at *t* = 5 s and after 5 s.

Figure 44 and Figure 45 show the error trajectory generated while reference tracking, the performance of the controller before and after addition of the constant disturbance, concludes the superiority of ADRC-NLSEF-TD over other controllers of trajectory tracking before and after addition of constant disturbance, under the influence of noise, with parameter variation.

Case 4: Harmonic Disturbance

**Figure 46:**
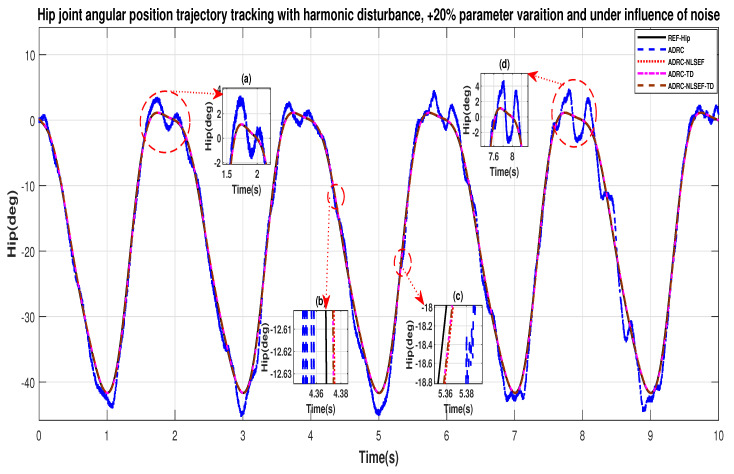
Gait trajectory tracking comparison of ADRC, ADRC-NLSEF, ADRC-TD, and ADRC-NLSEF-TD for the knee joint under the influence of noise, with parameter variation and with harmonic disturbance effect.

**Figure 47:**
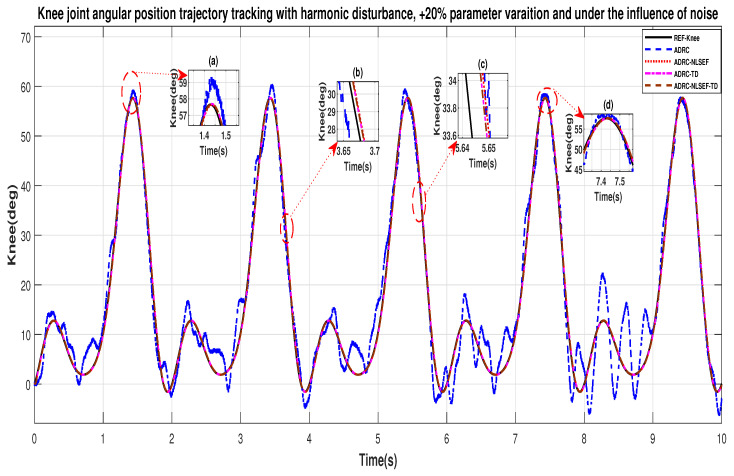
Gait trajectory tracking comparison of ADRC, ADRC-NLSEF, ADRC-TD, and ADRC-NLSEF-TD for the knee joint under the influence of noise, with parameter variation and with harmonic disturbance effect.

**Figure 48:**
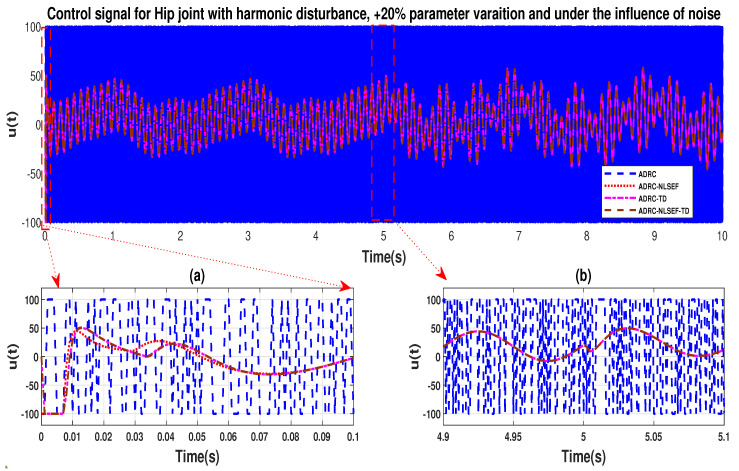
Control signal trajectory tracking comparison of ADRC, ADRC-NLSEF, ADRC-TD, and ADRC-NLSEF-TD for the knee joint under the influence of noise, with parameter variation and with harmonic disturbance effect.

**Figure 49:**
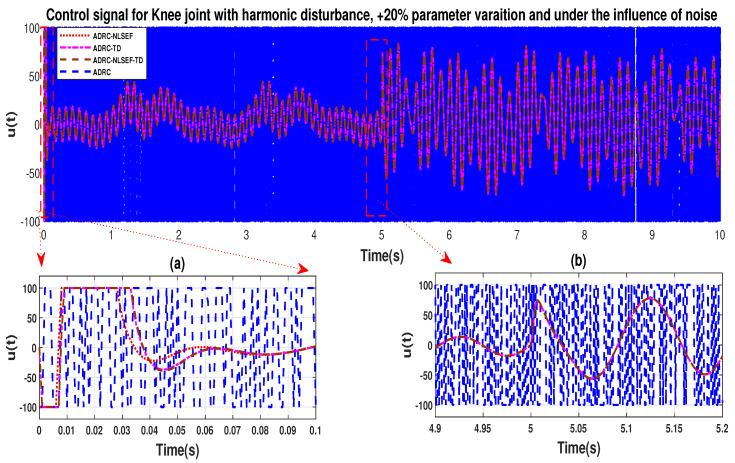
Control signal trajectory tracking comparison of ADRC, ADRC-NLSEF, ADRC-TD, and ADRC-NLSEF-TD for the knee joint under the influence of noise, with parameter variation and with harmonic disturbance effect.

**Figure 50:**
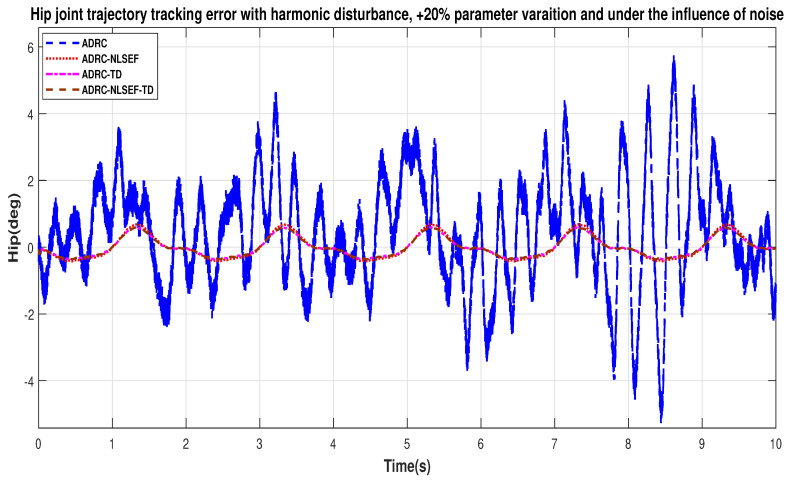
Gait trajectory tracking error comparison of ADRC, ADRC-NLSEF, ADRC-TD, and ADRC-NLSEF-TD for the hip joint under the influence of noise, with parameter variation and with harmonic disturbance effect.

**Figure 51:**
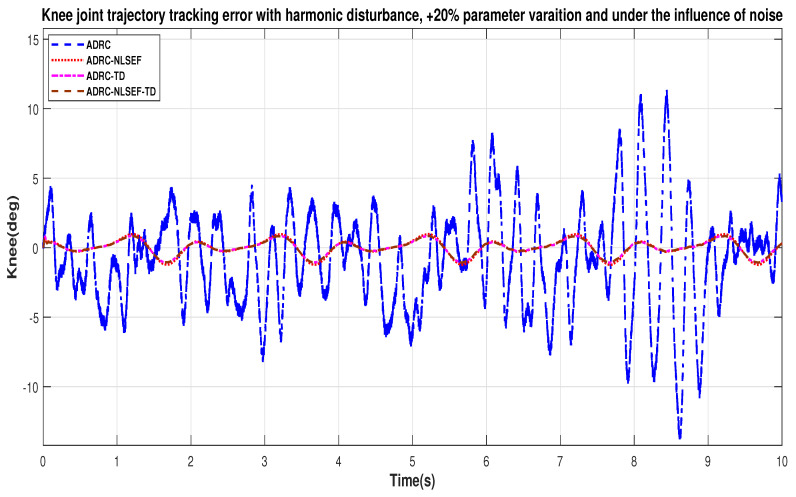
Gait trajectory tracking error comparison of ADRC, ADRC-NLSEF, ADRC-TD, and ADRC-NLSEF-TD for the hip joint under the influence of noise, with parameter variation and with harmonic disturbance effect.

In case 4, the performance of the ADRC is compared with the proposed controllers, under the influence of noise, with parameter variation with addition of harmonic control disturbance of amplitude *5 N.m.* at *t* = 5 s. Figure 46 and Figure 47 show the trajectory tracking performance for various controllers. Figure 48 and Figure 49 show control signal required. Figure 50 and Figure 51 show tracking error for ADRC, ADRC-NLSEF, ADRC-TD, and ADRC-NLSEF-TD for the hip and knee joints with harmonic disturbance.

In Figure 46 and Figure 47 show the trajectory tracking performance for various controllers. The minimized plot (a) and plot (b) show the trajectory tracking response of the controllers before the introduction of the harmonic disturbance. Plot (c) and plot (d) show the controller trajectory tracking performance after addition of harmonic disturbance at *t* = 5 s. The trajectory tracked by ADRC-NLSEF-TD has the best reference tracking followed by ADRC-TD, ADRC-NLSEF, and ADRC before and after inclusion of disturbance at *t* = 5 s, which proves its effectiveness under the influence of noise, with parameter variation and against harmonic disturbance.

The initial response of the control signal is shown in the minimized plot (a) and control signal in blown up in the minimized plot (b) shown in Figure 48 and Figure 49, gives us the idea of control signal required by the controllers. The control signal for ADRC completely saturates whereas in the proposed controlled methods it firstly saturates for a while and maintains its safe limit. Plot (b) shows the effect of harmonic disturbance at *t* = 5 s and after 5 s.

Figure 50 and Figure 51 show the error trajectory generated while reference tracking, the performance of the controller before and after addition of the constant disturbance, concludes the superiority of ADRC-NLSEF-TD over other controllers of trajectory tracking before and after addition of harmonic disturbance, under the influence of noise, with parameter variation.

The parametric variations of +20%, under the influence of noise and inclusion of disturbances over the ADRC, ADRC-NLSEF, ADRC-TD, and ADRC-NLSEF-TD controller resulted in the superior performance of ADRC-NLSEF-TD amongst all, for the gait trajectory tracking for the hip and knee joints, based on the performance indices. LESO based ADRC fails to track the trajectory and is severely affected by noise which can be clearly visible through the trajectory tracking, control signal, and large tracking error. Performance shown in the figures and based on performance indices, the proposed control method performs superior and tracks the trajectory efficiently as compared to ADRC even with parameter variation, under the effect of noise and disturbance and with actuator saturation it keeps the control signal in safe operation limits.

## 7. Discussion

The use of exoskeleton has various potential applications in the medical and non-medical fields. A medical exoskeleton is utilized for rehabilitation over conventional methods for better treatment, again it is useful for the persons suffering from the loss of limbs (amputees) to provide mobility. In non-medical, it can be utilized to support human workers in industries for physically demanding tasks such as heavy load lifting, for the soldiers in wartime or medical emergencies exoskeleton is helpful for strength augmentation, and elderly persons to perform the daily chores by providing reduced physical effort, its application has already started in some countries.

This paper is more focused on designing the control based on the passive rehabilitation aspect of given exoskeleton for gait tracking. The use of exoskeletons for rehabilitation requires special care as the motion trajectories for joints can not be provided through the wearer, the affected person cannot make the required actions. The international safety regulatory requirements (published by the International Organization for Standardization (ISO; www.iso.org) and International Electro-technical Commission (IEC; www.iec.ch)) for medical exoskeletons, such regulations are still underdeveloped by the joint working group IEC SC62D and ISO TC299 JWG36 (medical robots for rehabilitation). The design and development of the algorithm for the lower limb rehabilitation robotic devices was the preliminary task. The experimental work for the proposed system includes testing of all combinations of ADRC algorithm presented in this paper with LESO based ADRC. In future experimentation, the protocol and set up will be undertaken in a controlled laboratory environment. Each volunteer performs two trials of a 5 gait cycle for each controller. During trials the exoskeleton wearer will walk forward. The exoskeleton will be connected to the wearer’s lower limb through the connection cuff, the walking cycle is tested on flat terrain. The control enclosure will have an embedded computer, the actuators, encoders, and power modules. The embedded pc will send the desired command signals to the actuators and then generates the control signal to drive the exoskeleton to follow the predefined gait. The encoder will capture the angular position of the joints and send back to the embedded pc.

In this paper, an I-ADRC method is proposed which is an extension to the work [39]. The paper compares various combinations of ADRC with the LESO based ADRC [39], it can be concluded from the results that I-ADRC has improved trajectory tracking response, better performance is obtained under the influence of noise and disturbance, again it gives an improved performance with parameter variation, although the proposed method has some disadvantages over ADRC that the design becomes complex and number of tuning parameters increases. The proposed method is found to be more accurate for the given modeled system but its scope is not limited. The proposed method developed in this paper specifically addresses the rehabilitation issue, the proposed algorithm is the generalized method and can be utilized for the areas of interest.

## 8. Conclusions

In this paper an improved active disturbance rejection control (I-ADRC) method encompasses of linear extended state observer (LESO), tracking differentiator (TD) and nonlinear state error feedback (NLSEF) is effectively applied for sagittal plane gait trajectory tracking on a 2 DoF LLRRE with the hip and the knee joints in the simulation study. The performance indices ITSE, ITAE, IAE, ISE, and ISU reflects the potential of proposed ADRC combinations over ADRC in terms of trajectory tracking, control signal requirement, and disturbance rejection, under the influence of noise and parametric variation. Amongst all the controllers, ADRC-NLSEF-TD proves a better selection because of its best tracking capabilities followed by ADRC-TD and ADRC-NLSEF, which proves it as a promising strategy. Proposed ADRC in the future can be used for various assistive devices/exoskeletons and orthoses for improvement in tracking. This article presented the overall results of the controller with emphasis on simulation. In the next phase of research in the coming months, it would be validated through experimental work.

## Figures and Tables

**Table 1:** Parameters of the exoskeleton.

Parameter	Symbol	Numerical Value
Thigh segment	$m_{h}$	5 kg
Length of thigh	$l_{h}$	435 mm
Length of shank	$l_{k}$	475 mm
Shank segment	$m_{k}$	2 kg
Gravity constant	*g*	9.81 m/s${}^{2}$

**Table 2:** Coefficients for the equation.

Coefficient	Value	Coefficient	Value
c${}_{0}$	0.208	f${}_{1}$	−0.103
c${}_{1}$	0.362	f${}_{2}$	−0.010
c${}_{2}$	−0.066	f${}_{3}$	0.029
c${}_{3}$	0.001	f${}_{4}$	−0.342
c${}_{4}$	0.766	f${}_{5}$	0.168
c${}_{5}$	−0.099	f${}_{6}$	0.084
c${}_{6}$	−0.219	d	3.142
c${}_{7}$	0.008	d${}_{1}$	3.142

**Table 3:** The control law for proposed active disturbance rejection control (ADRC) with combinations

Controller	Control Law
ADRC	$\begin{array}{c} U={\left[\tau_{1},\tau_{2}\right]}^{T}=D_{inv}\left(K_{p}e+K_{d}\dot{e}-\overset{\wedge }{f}\right)\\ for\;hip\;joint\;e=\left(q_{h,d}-\overset{\wedge }{q_{1}}\right),\dot{e}=\overset{\wedge }{q_{2}};\;\overset{\wedge }{f}=\overset{\wedge }{q_{3},}\\ for\;knee\;joint\;e=\left(q_{k,d}-\overset{\wedge }{q_{4}}\right),\dot{e}=\overset{\wedge }{q_{5}};\;\overset{\wedge }{f}=\overset{\wedge }{q_{6},} \end{array}$
ADRC-NLSEF	$\begin{array}{c} \begin{array}{c} U={\left[\tau_{1},\tau_{2}\right]}^{T}=D_{inv}\left(K_{p}\;\phi \left(e,\alpha ,\delta \right)-K_{d}\;\phi \left(\dot{e},\alpha ,\delta \right)\;-\overset{\wedge }{f}\right)\\ where,\;for\;hip\;joint\;,e=\left(q_{h,d}-\phi \left(\overset{\wedge }{q_{1}},\alpha ,\delta \right)\right),\dot{e}=\phi \left(\overset{\wedge }{q_{2}},\alpha ,\delta \right);\;\overset{\wedge }{f}=\overset{\wedge }{q_{3},}\\ for\;knee\;joint\;,e=\left(q_{k,d}-\phi \left(\overset{\wedge }{q_{4}},\alpha ,\delta \right)\right),\dot{e}=\phi \left(\overset{\wedge }{q_{5}},\alpha ,\delta \right);\;\overset{\wedge }{f}=\overset{\wedge }{q_{6},} \end{array} \end{array}$
ADRC-TD	$\begin{array}{c} \begin{array}{c} U={\left[\tau_{1},\tau_{2}\right]}^{T}=D_{inv}\left(K_{p}e+K_{d}\dot{e}\;-\overset{\wedge }{f}\right)\\ where,\;for\;hip\;joint\;e=\left(q_{k,d}-\overset{\wedge }{q_{1}}\right),\dot{e}=\left(\dot{q_{d}}-\overset{\wedge }{q_{2}}\right),\;\overset{\wedge }{f}=\overset{\wedge }{q_{3},} \end{array}\\ for\;knee\;joint\;e=\left(q_{h,d}-\overset{\wedge }{q_{4}}\right),\dot{e}=\left(\dot{q_{d}}-\overset{\wedge }{q_{5}}\right),\;\overset{\wedge }{f}=\overset{\wedge }{q_{6},} \end{array}$
ADRC-NLSEF-TD	$\begin{array}{c} \begin{array}{c} \begin{array}{c} U={\left[\tau_{1},\tau_{2}\right]}^{T}=D_{inv}\left(K_{p}\;\phi \left(e,\alpha ,\delta \right)+K_{d}\dot{e}-\overset{\wedge }{f}\right)\\ where,\;for\;hip\;joint\;e=(q_{h,d}-\overset{\wedge }{q_{1}}),\; \end{array}\\ \dot{e}=\left(\dot{q_{h,d}}-\phi \left(\overset{\wedge }{q_{2}},\alpha ,\delta \right)\right),\;\overset{\wedge }{f}=\overset{\wedge }{q_{3},}\\ for\;hip\;joint\;e=(q_{k,d}-\overset{\wedge }{q_{4}}),\;\\ \dot{e}=\left(\dot{q_{k,d}}-\phi \left(\overset{\wedge }{q_{5}},\alpha ,\delta \right)\right),\;\overset{\wedge }{f}=\overset{\wedge }{q_{6},} \end{array} \end{array}$

**Table 4:** The parameters selection for tracking differentiator.

Parameters	Variation	Final Selected Values
*a${}_{1}$*	5 *to 50*	*30*
*b*${}_{1}$ and *b*${}_{2}$	*1 to 10*	*5*
*R*	*10 to 80*	*30*
*n*	*1, 3, and 5*	*3*

**Table 5:** The parameters selection for NLSEF.

Parameters	Variation	Final Selected Values
α	*0.5 to 1*	0.995
δ	*0.001 to 0.5*	0.01

**Table 6:** Performance indices for ADRC, ADRC-NLSEF, ADRC-TD, and ADRC-NLSEF-TD for the hip joint and the knee joint for the no disturbance case.

Control Method		ADRC-NLSEF-TD		ADRC-TD		ADRC-NLSEF		ADRC	
Joints		Hip	Knee	Hip	Knee	Hip	Knee	Hip	Knee
Performance indices	ITSE (Deg.)	4.241	13.2	4.253	13.32	5.793	17.95	6.083	18.87
	ISE (Deg.)	0.8447	2.454	0.8468	2.477	1.152	3.332	1.209	3.503
	ITAE (Deg.)	11.85	20.3	11.85	20.36	13.86	23.68	14.19	24.27
	IAE (Deg.)	2.397	3.883	2.397	3.895	2.8	4.526	2.866	4.638
	ISU N.m.)$\times 10^{4}$	0.1292	0.1739	0.1292	0.1738	0.1293	0.1724	0.1239	0.1722

**Table 7:** Performance indices for ADRC, ADRC-NLSEF, ADRC-TD, and ADRC-NLSEF-TD for the hip joint and the knee joint for random disturbance case.

Control Method		ADRC-NLSEF-TD		ADRC-TD		ADRC-NLSEF		ADRC	
Joints		Hip	Knee	Hip	Knee	Hip	Knee	Hip	Knee
Performance indices	ITSE (Deg.)	4.241	13.2	4.253	13.32	5.793	17.95	6.082	18.87
	ISE (Deg.)	0.8447	2.454	0.8468	2.477	1.152	3.332	1.209	3.503
	ITAE (Deg.)	11.85	20.3	11.85	20.36	13.86	23.68	14.19	24.27
	IAE (Deg.)	2.397	3.883	2.397	3.895	2.8	4.526	2.866	4.638
	ISU (N.m.)$\times 10^{4}$	0.1298	0.1817	0.1298	0.1817	0.1299	0.1803	0.1299	0.1801

**Table 8:** Performance indices for ADRC, ADRC-NLSEF, ADRC-TD, and ADRC-NLSEF-TD for the hip joint and the knee joint for the constant disturbance case.

Control Method		ADRC-NLSEF-TD		ADRC-TD		ADRC-NLSEF		ADRC	
Joints		Hip	Knee	Hip	Knee	Hip	Knee	Hip	Knee
Performance indices	ITSE (Deg.)	4.241	13.21	4.252	13.34	5.792	17.96	6.081	18.89
	ISE (Deg.)	0.8446	2.456	0.8466	2.478	1.152	3.334	1.209	3.505
	ITAE (Deg.)	11.85	20.31	11.85	20.37	13.86	23.69	14.19	24.28
	IAE (Deg.)	2.397	3.884	2.398	3.896	2.8	4.527	2.866	4.639
	ISU (N.m.)$\times 10^{4}$	0.6217	3.167	0.6217	3.167	0.6214	3.166	0.6214	3.166

**Table 9:** Performance indices for ADRC, ADRC-NLSEF, ADRC-TD, and ADRC-NLSEF-TD for the hip joint and the knee joint for harmonic disturbance case.

Control Method		ADRC-NLSEF-TD		ADRC-TD		ADRC-NLSEF		ADRC	
Joints		Hip	Knee	Hip	Knee	Hip	Knee	Hip	Knee
Performance indices	ITSE (Deg.)	4.243	13.22	4.255	13.34	5.795	17.97	6.085	18.89
	ISE (Deg.)	0.845	2.457	0.847	2.48	1.152	3.335	1.21	3.506
	ITAE (Deg.)	11.86	20.35	11.86	20.41	13.87	23.73	14.2	24.32
	IAE (Deg.)	2.399	3.89	2.399	3.902	2.801	4.533	2.867	4.645
	ISU (N.m.)$\times 10^{4}$	0.3034	2.039	0.3035	2.041	0.3035	2.038	0.3037	2.039

**Table 10:** Overall performance indices the hip joint.

Hip Joint						
Control Method	Disturbance Case	ITSE (Deg.)	ISE (Deg.)	ITAE (Deg.)	IAE (Deg.)	ISU (N.m.) $\times 10^{4}$
ADRC-NLSEF-TD	Case 1	4.241	0.8447	11.85	2.397	0.1292
	Case 2	4.241	0.8447	11.85	2.397	0.1298
	Case 3	4.241	0.8446	11.85	2.397	0.6217
	Case 4	4.243	0.8450	11.86	2.399	0.3034
ADRC-TD	Case 1	4.253	0.8468	11.85	2.397	0.1292
	Case 2	4.253	0.8468	11.85	2.397	0.1298
	Case 3	4.252	0.8466	11.85	2.398	0.6217
	Case 4	4.255	0.8470	11.86	2.399	0.3035
ADRC-NLSEF	Case 1	5.793	1.152	13.86	2.800	0.1293
	Case 2	5.793	1.152	13.86	2.800	0.1299
	Case 3	5.792	1.152	13.86	2.800	0.6214
	Case 4	5.795	1.152	13.87	2.801	0.3035
ADRC	Case 1	6.083	1.209	14.19	2.866	0.1293
	Case 2	6.082	1.209	14.19	2.866	0.1299
	Case 3	6.081	1.209	14.19	2.866	0.6214
	Case 4	6.085	1.21	14.20	2.867	0.3037

Case1: no disturbance, Case2: random disturbance, Case 3: constant disturbance, Case 4: harmonic disturbance.

**Table 11:** Overall performance indices for the knee joint.

Knee Joint						
Control Method	Disturbance Case	ITSE (Deg.)	ISE (Deg.)	ITAE (Deg.)	IAE (Deg.)	ISU (N.m.) $\times 10^{4}$
ADRC-NLSEF-TD	Case 1	13.20	2.454	20.30	3.883	0.1739
	Case 2	13.20	2.454	20.30	3.883	0.1818
	Case 3	13.21	2.456	20.31	3.884	3.167
	Case 4	13.22	2.457	20.35	3.890	2.039
ADRC-TD	Case 1	13.32	2.477	20.36	3.895	0.1738
	Case 2	13.32	2.477	20.36	3.895	0.1817
	Case 3	13.34	2.478	20.37	3.896	3.167
	Case 4	13.34	2.48	20.41	3.902	2.041
ADRC-NLSEF	Case 1	17.95	3.332	23.68	4.526	0.1724
	Case 2	17.95	3.332	23.68	4.526	0.1803
	Case 3	17.96	3.334	22.69	4.527	3.166
	Case 4	17.97	3.335	22.73	4.533	2.038
ADRC	Case 1	18.87	3.503	24.27	4.638	0.1722
	Case 2	18.87	3.503	24.27	4.638	0.1801
	Case 3	18.89	3.505	24.28	4.639	3.166
	Case 4	18.89	3.506	27.32	4.645	2.039

Case1: no disturbance, Case 2: random disturbance, Case 3: constant disturbance, Case 4: harmonic disturbance.

**Table 12:** Parameters of the exoskeleton.

Parameter	Symbol	Numerical Value (Actual)	−20% Varied Values	20% Varied Values
Thigh segment	$m_{h}$	5 kg	4 Kg	6 kg
length of thigh	$l_{h}$	435 mm	348 mm	522 mm
Length of shank	$l_{k}$	475 mm	380 mm	570 mm
Shank segment	$m_{k}$	2 kg	1.6 kg	2.4 kg

**Table 13:** Overall performance indices of the hip joint ±20% parameter variation.

Hip Joint											
		ITSE (Deg.)		ISE (Deg.)		ITAE (Deg.)		IAE (Deg.)		ISU (N.m.) $\times 10^{4}$	
Control Method	Disturbance Case	−20%	+20%	−20%	+20%	−20%	+20%	−20%	+20	−20%	+20%
ADRC-TD-NLSEF	Case 1	4.240	4.242	0.8444	0.8448	11.85	11.85	2.397	2.397	0.1829	0.09875
	Case 2	4.240	4.242	0.8444	0.8448	11.85	11.85	2.397	2.397	0.1850	0.09897
	Case 3	4.238	4.241	0.8441	0.8447	11.85	11.85	2.397	2.398	1.848	0.2869
	Case 4	4.252	4.243	0.846	0.8449	11.88	11.86	2.401	2.398	0.8465	0.1572
ADRC-TD	Case 1	4.251	4.253	0.8464	0.8468	11.85	11.85	2.397	2.398	0.1829	0.09875
	Case 2	4.251	4.253	0.8464	0.8468	11.85	11.85	2.397	2.398	0.1850	0.09897
	Case 3	4.249	4.252	0.8461	0.8467	11.85	11.85	2.397	2.398	1.848	0.2869
	Case 4	4.264	4.254	0.8481	0.847	11.88	11.86	2.401	2.398	0.8470	0.1572
ADRC-NLSEF	Case 1	5.791	5.793	1.152	1.152	13.85	13.86	2.799	2.8	0.1830	0.09894
	Case 2	5.791	5.793	1.152	1.152	13.85	13.86	2.799	2.8	0.1851	0.09916
	Case 3	5.789	5.793	1.151	1.152	13.86	13.86	2.799	2.8	1.848	0.2871
	Case 4	5.803	5.794	1.153	1.152	13.88	13.86	2.803	2.8	0.8467	0.1574
ADRC	Case 1	6.081	6.083	1.209	1.21	14.19	14.19	2.866	2.866	0.1830	0.09892
	Case 2	6.081	6.083	1.209	1.21	14.19	14.19	2.866	2.866	0.1851	0.09914
	Case 3	6.078	6.082	1.209	1.209	14.19	14.19	2.866	2.866	1.848	0.2871
	Case 4	6.093	6.084	1.211	1.21	14.22	14.19	2.87	2.867	0.8472	0.1574

Case1: no disturbance, Case 2: random disturbance, Case 3: constant disturbance, Case 4: harmonic disturbance.

**Table 14:** Overall performance indices of the knee joint ±20% parameter variation.

Knee Joint											
		ITSE (Deg.)		ISE (Deg.)		ITAE (Deg.)		IAE (Deg.)		ISU (N.m.) $\times 10^{4}$	
Control Method	Disturbance Case	−20%	+20%	−20%	+20%	−20%	+20%	−20%	+20	−20%	+20%
ADRC-TD-NLSEF	Case 1	13.20	13.2	2.456	2.454	20.3	20.3	3.885	3.883	0.2489	0.1362
	Case 2	13.21	13.2	2.456	2.454	20.3	20.3	3.885	3.883	0.2759	0.1392
	Case 3	13.24	13.21	2.459	2.455	20.32	20.3	3.886	3.884	12.76	1.009
	Case 4	13.31	13.21	2.47	2.455	20.44	20.33	3.903	3.888	7.372	0.7600
ADRC-TD	Case 1	13.33	13.32	2.478	2.477	20.37	20.36	3.896	3.895	0.2489	0.1361
	Case 2	13.33	13.32	2.478	2.477	20.37	20.36	3.896	3.895	0.2757	0.1391
	Case 3	13.36	13.33	2.482	2.478	20.39	20.37	3.898	3.895	12.76	1.009
	Case 4	13.44	13.33	2.493	2.478	20.51	20.4	3.916	3.899	7.377	0.7604
ADRC-NLSEF	Case 1	17.96	17.95	3.333	3.332	23.69	23.68	4.527	4.526	0.2475	0.1347
	Case 2	17.96	17.95	3.333	3.332	23.69	23.68	4.527	4.526	0.2744	0.1377
	Case 3	17.99	17.96	3.338	3.333	23.71	23.69	4.529	4.526	12.76	1.008
	Case 4	18.07	17.96	3.348	3.333	23.83	23.72	4.546	4.531	7.372	0.7586
ADRC	Case 1	18.88	18.87	3.504	3.503	24.28	24.27	4.639	4.638	0.2473	0.1345
	Case 2	18.88	18.87	3.504	3.503	24.27	24.27	4.639	4.638	0.2741	0.1375
	Case 3	18.92	18.88	3.509	3.504	24.29	24.27	4.461	4.638	12.76	1.007
	Case 4	18.99	18.88	3.52	3.504	24.42	24.31	4.658	4.642	7.376	0.7588

Case1: no disturbance, Case 2: random disturbance, Case 3: constant disturbance, Case 4: harmonic disturbance.

**Table 15:** Overall performance indices of the hip joint +20% parameter variation and under influence of noise.

Hip Joint						
Control Method	Disturbance Case	ITSE (Deg.)	ISE (Deg.)	ITAE (Deg.)	IAE (Deg.)	ISU (N.m.) $\times 10^{4}$
ADRC-TD-NLSEF	Case 1	4.244	0.8461	11.87	2.404	0.7248
	Case 2	4.244	0.8461	11.87	2.404	0.7277
	Case 3	4.243	0.8460	11.87	2.404	0.9132
	Case 4	4.245	0.8462	11.87	2.404	0.7802
ADRC-TD	Case 1	4.256	0.8482	11.87	2.404	0.7252
	Case 2	4.256	0.8482	11.87	2.404	0.7281
	Case 3	4.255	0.8481	11.87	2.404	0.9135
	Case 4	4.257	0.8483	11.87	2.405	0.7806
ADRC-NLSEF	Case 1	5.796	1.153	13.87	2.805	0.7239
	Case 2	5.796	1.153	13.87	2.805	0.7269
	Case 3	5..795	1.153	13.87	2.805	0.9123
	Case 4	5.797	1.153	13.88	2.805	0.7794
ADRC	Case 1	144	25.03	67.94	12.69	9.914
	Case 2	150.7	25.79	68.39	12.73	9.906
	Case 3	380.5	56.62	110.5	18.38	9.914
	Case 4	173.9	29.17	73.35	13.48	9.912

Case1: no disturbance, Case 2: random disturbance, Case 3: constant disturbance, Case 4: harmonic disturbance.

**Table 16:** Overall performance indices of the Knee joint +20% parameter variation and under influence of noise.

Knee Joint						
Control Method	Disturbance Case	ITSE (Deg.)	ISE (Deg.)	ITAE (Deg.)	IAE (Deg.)	ISU (N.m.) $\times 10^{4}$
ADRC-TD-NLSEF	Case 1	13.20	2.464	20.31	3.894	0.8040
	Case 2	13.20	2.464	20.31	3.894	0.8240
	Case 3	13.21	2.465	20.32	3.894	1.659
	Case 4	13.21	2.465	20.34	3.898	1.44
ADRC-TD	Case 1	13.32	2.486	20.38	3.905	0.8031
	Case 2	13.33	2.486	20.38	3.905	0.8230
	Case 3	13.33	2.487	20.38	3.906	1.658
	Case 4	13.34	2.488	20.41	3.910	1.439
ADRC-NLSEF	Case 1	17.95	3.338	23.70	4.535	0.7989
	Case 2	17.95	3.338	23.70	4.535	0.8189
	Case 3	17.96	3.339	23.71	4.536	1.655
	Case 4	17.96	3.340	23.73	4.540	1.434
ADRC	Case 1	734.7	121.7	146.4	27.1	9.716
	Case 2	776.1	126.3	149.3	27.4	9.730
	Case 3	2149	308.7	253	41.37	9.742
	Case 4	873.6	140.7	157.2	28.78	9.742

Case1: no disturbance, Case 2: random disturbance, Case 3: constant disturbance, Case 4: harmonic disturbance.
